# The two-directional prospective association between inflammatory bowel disease and neurodegenerative disorders: a systematic review and meta-analysis based on longitudinal studies

**DOI:** 10.3389/fimmu.2024.1325908

**Published:** 2024-04-24

**Authors:** Jiahao Zong, Yue Yang, Hui Wang, Huipeng Zhang, Xiaorong Yang, Xiaoyun Yang

**Affiliations:** ^1^ Department of Gastroenterology, Qilu Hospital of Shandong University, Jinan, China; ^2^ Shandong Provincial Clinical Research Center for Digestive Disease, Shandong, China; ^3^ Laboratory of Translational Gastroenterology, Qilu Hospital of Shandong University, Jinan, Shandong, China; ^4^ Department of General Surgery, Xinhua Hospital, Shanghai Jiao Tong University School of Medicine, Shanghai, China; ^5^ Clinical Epidemiology Unit, Clinical Research Center of Shandong University, Qilu Hospital of Shandong University, Jinan, Shandong, China

**Keywords:** inflammatory bowel disease, neurodegenerative disorder, meta-analysis, longitudinal studies, Parkinson’s disease, dementia, multiple sclerosis, Alzheimer’s disease

## Abstract

**Objective:**

Previous studies reported possible connections between inflammatory bowel disease (IBD) and several neurodegenerative disorders. However, the comprehensive relationships between IBD and various neurodegenerative disorders were not summarized. We executed a meta-analysis of longitudinal studies to provide an estimate of the strength of the two-directional prospective association between IBD and neurodegenerative disorders.

**Methods:**

We accomplished a thorough bibliographic search of PubMed, Web of Science, Embase, PsycINFO, and Cochrane Library databases until June 2023 to locate relevant longitudinal studies. The extracted data were then analyzed via meta-analysis using either a fixed or random effects model.

**Results:**

The final analysis encompassed 27 studies. Individuals with IBD faced an increased risk of developing four neurodegenerative disorders than the general public, namely, Alzheimer’s disease (hazard ratio[HR] = 1.35, 95% confidence interval [CI]: 1.03–1.77, P=0.031), dementia (HR =1.24, 95% CI: 1.13–1.36, P<0.001), multiple sclerosis (HR =2.07, 95% CI:1.42–3.02, P<0.001) and Parkinson’s disease (HR =1.23, 95% CI:1.10–1.38, P<0.001). Two articles reported an increased incidence of amyotrophic lateral sclerosis or multiple system atrophy in IBD patients. Three studies investigated the prospective association between multiple sclerosis and IBD, revealing an elevated risk of the latter in patients with the former. (HR=1.87, 95% CI:1.66–2.10, P<0.001).

**Interpretation:**

These findings verified the two-directional relationship between the brain-gut axis, specifically demonstrating a heightened risk of various neurodegenerative diseases among IBD patients. It may be profitable to prepare screening strategies for IBD patients to find neurodegenerative diseases during the long-term course of treatment for IBD with a view to potential earlier diagnosis and treatment of neurodegenerative diseases, reducing public health and social burden.

**Systematic Review Registration:**

PROSPERO (CRD42023437553).

## 1 Introduction

Neurodegenerative disorders consist of a group of chronic central nervous system disorders with heterogeneity, including Alzheimer’s disease (AD), dementia with Lewy bodies (DLB), Parkinson’s disease (PD), multiple sclerosis (MS), multiple system atrophy (MSA), progressive supranuclear palsy (PSP), Huntington’s disease (HD), etc., whose primary feature is a progressive loss of neurons. They are a major burden for our society, affecting millions of people worldwide (1). These patients need ongoing and long-term care, which is associated with significant economic and social costs. Prince et al. estimated that by 2030, the global cost of dementia alone will surpass $2 trillion by 2030 (2). As the world population ages and life expectancy increases, early diagnosis and treatment of neurodegenerative diseases have become a worldwide public health issue. On the other hand, inflammatory bowel disease (IBD) represents a long-standing inflammatory disorder of the intestines, encompassing Crohn’s disease (CD) and ulcerative colitis (UC), whose exact triggering factors are not yet identified (3). With the prevalence on the rise, IBD poses massive financial stress on public healthcare systems and presents a global healthcare challenge (1, 4).

There is an expanding body of evidence for two-directional regulation between gastrointestinal tract disorders and central nervous system dysfunction, generally stated as the “gut-brain axis” theory (5). The disturbance of gut bacteria and chronic intestinal inflammation may lead to systemic inflammatory responses which damage the blood-brain barrier, stimulate the neuroinflammation process, and finally increase the incidence of neurodegenerative diseases (6). To date, several erstwhile meta-analyses have investigated the relationship between IBD and the incidence of one specific neurodegenerative disease (7–10). These studies suggested a prospective connection between IBD and neurodegenerative diseases, indicating a possible prospective association. However, most of their studies were not limited to the types of studies included, results from cross-sectional, case-control and longitudinal studies were often pooled. Given the inherent inadequacies of case-control study design, which requires retrospective collection of potential exposure risk factors, neurodegenerative diseases can greatly affect the accuracy of reporting. Moreover, cross-sectional and case-control studies usually involve selection bias and other measurement bias, therefore, we focused on longitudinal studies to obtain more reliable results, whose design allows temporality to be established by assessing exposure before disease onset. In the current study, we undertook a meta-analysis of higher-quality longitudinal studies from different countries to sum up existing studies on IBD and all neurodegenerative diseases and examine the two-directional link between IBD and the risk of neurodegeneration.

## 2 Methods

This systematic review and meta-analysis abided by the Preferred Reporting Items for Systematic Reviews and Meta-Analyses (PRISMA 2020) guidelines (11). The study strategy was recorded beforehand within the PROSPERO platform (registration number: CRD42023437553).

### 2.1 Search strategy and selection criteria

We accomplished a comprehensive bibliographic search of PubMed, Web of Science, Embase, PsycINFO, and Cochrane Library up to 18 June 2023 to identify cohort studies investigating the prospective correlation between IBD and neurodegenerative disorders. Search regulations were formulated built on Medical Subject Headings index words (such as inflammatory bowel diseases, neurocognitive disorders, cohort studies) and free words (such as ulcerative colitis, Crohn, Alzheimer, Parkinson, dementia, and multiple sclerosis). No language restriction was imposed on the search. Articles that possibly assessed the prevalence or incidence of neurodegenerative diseases or IBD were selected first by the title, and then by the abstract. Two independent investigators (HW and H-PZ) conducted the search and data abstraction. To potentially identify eligible studies, the reference indexes of included articles and relevant review papers were estimated and searched manually. **Supplementary File 1** contains our elaborate search strategies for each bibliography database.

The included criteria for our meta-analysis were identified in such a way: (1) Documents that reported the analysis results between neurodegenerative diseases and IBD encompassing UC, CD and IBD-unclassified (IBD-U); (2) Documents that employed a longitudinal research design; (3) Documents that reported estimates of crude or adjusted epidemiological effects, such as hazard ratio (HR), incidence rate ratio (IRR), odds ratio (OR), risk ratio (RR), or standardized incidence ratio (SIR), along with a parallel 95% confidence interval (CI), or data could be obtained to calculate the aforementioned effect estimates; (4) Documents in which IBD and neurodegenerative diseases must be defined or clinically diagnosed abiding by the International Classification of Diseases (ICD) or standard diagnostic criteria. Participants in this meta-analysis were not restricted by sex, age, ethnicity, race, or comorbidities.

Criteria for exclusion were listed in such a way: (1) Conference abstracts, case reports, case series, ecologic studies, editorials, various reviews, practice guidelines, cross-sectional studies, and case-control studies; (2) Studies with a duration of follow-up less than 1 year; (3) Studies that did not accurately provide any effect estimates or 95% CIs for the outcome of our interests.

### 2.2 Data extraction and quality assessment

Two researchers (HW and H-PZ) conducted an independent evaluation and analysis on all titles and abstracts of the search records against the inclusion and exclusion criteria. Conceivably qualifying documents were subsequently evaluated through reading full-text after which justifications for exclusion were recorded. Any disagreements were settled by referring back to the original document and the collective consensus was reached in consultation with the third author (YY). Data were excavated from the final included documents including the first author, study population, the type of neurodegenerative disease, age, and sample size. In instances of duplicate publications, we have nominated the most comprehensive or up-to-date information available. All our research data was recorded and summarized by one author (HW) and verified by another (H-PZ).

Two authors (HW and H-PZ) conducted independent evaluations of potential biases. Any inconsistencies were resolved via reassessment by a third author (YY) of the original article. The research quality of these included studies was evaluated by applying the Newcastle-Ottawa Scale (NOS), since all studies were non-randomized and had a longitudinal design strategy (12). Three domains of a study are assessed by this scale, which are participants’ representativeness, equivalence of different exposure groups characteristics, and identification of research outcomes of interest, with a star scale that tops at 9 stars. Studies scoring 9 stars were adjudicated to be at minuscule risk of bias, those scoring 7 or 8 stars at moderate risk, and those scoring ≤6 stars at nonnegligible risk of bias.

### 2.3 Data analysis

The primary outcome was the occurrence rate of neurodegenerative disorders in IBD patients compared with IBD-free controls or the occurrence rate of IBD in patients with neurodegenerative disorders in comparison with controls without neurodegenerative disorders. The effect estimates would be excavated for studies that provide both unadjusted and adjusted risk estimates. The HRs, ORs, RRs or SIRs were considered equivalent because the incidence of neurodegenerative diseases was relatively low. The HRs (or ORs, RRs and SIRs) and 95% CIs were regarded as the association values for every ultimately included study. For studies reporting effect size with diverse degrees of covariate adjustment, we extracted those reflecting the maximal extent of possible confounding factors adjustment. Then we summarized the crude or adjusted HRs of all included studies, and calculated an interesting overall effect size with a fixed or random effects model.

In the current study, the entire data were analyzed by using Stata V.15.1. Statistical heterogeneity was approximately evaluated using the visual examination of the forest plots. We also assessed heterogeneity with the I²-statistics, which estimates the contribution of variability across included studies due to heterogeneity as opposed to the randomized factor. A rough guide to interpreting the data is as follows: I²-values of around 25% denote minuscule heterogeneity; approximately 50% denote moderate heterogeneity and around 75% denote nonnegligible heterogeneity (13). Whilst there was nonnegligible heterogeneity among the enrolled studies (I² > 50%), we utilized the random effect model to summarize the ultimate association. In contrast, we utilized the fixed effect model whilst heterogeneity was moderately low (I² < 50%). The funnel plot and the rank correlation Egger’s test were used to evaluate the risk of publication bias (14). To evaluate the sensitivity of our merged research results, we gradually removed one study at a time analysis and repeated the pooled process. Additionally, the robustness was assessed through a comparison of the outcomes from the random or fixed effect models. Based on age groups (<50, ≥50 years), IBD subtype (CD, UC), country (Europe, North America and Asia), and covariates adjustment (yes, no), subgroup analyses were performed.

## 3 Results

### 3.1 Features of all included studies

**Supplementary Figure 1** summarizes the gradual results of the article retrieval and study selection. On the basis of the titles and abstracts of 2291 citations, a total of 82 studies were primarily identified as potentially qualified from five electronic databases up to 18th June 2023. After careful examination of the full text, we ultimately included 27 in the meta-analysis (15–41). Twenty-four studies explored the risk of multifarious neurodegenerative diseases in people with IBD. The twenty-four included studies explored comprising at least 92,943,962 participants were all population-based and all studies were cohort studies. Population characteristics are summarized in **Table 1**. Fourteen studies were carried out in Europe (five in the UK, four in Danmark, three in Sweden, and two in Germany); five studies were carried out in America (four in the USA and one in Canada) and five studies were carried out in Asia (two in Taiwan, China and three in Korea). 17 of 24 studies adjusted or matched for possible confounders such as age, sex, and comorbidities. Details of each study are presented in **Table 1**. Three studies explored the risk of overall IBD in patients with neurodegenerative disorders. **Supplementary Table 1** contains comprehensive information for each study included in the final analysis.

**Table 1:** Characteristics of 25 studies exploring the risk of neurodegenerative diseases in patients with IBD.

study	disease	Country	Age (years)	Study period	follow-up time (mean)	Effect estimate, 95%CI		IBD	Concomitant disease	Non-IBD	Concomitant disease	NOS score
Aggarwal,2022 (15)	AD	USA	≥18	1999-2020	NA	IBD:2.30 (2.10-2.51) CD:3.34 (3.25-3.42) UC:1.09 (1.06-1.14)	OR	IBD (342,740) CD(191,530) UC(154,510)	AD(5,750) AD(2,500) AD(2,850)	64,857,840	AD(723,540)	9
Kim,2022 (16)	AD	Korea	55.4 ± 11.0†	2009-2017	6years	IBD:1.14 (1.05-1.25) CD:1.19 (1.00-1.41) UC:1.13 (1.02-1.24)	HR	IBD(24,830) CD(4,454) UC(20,376)	AD(644) AD(138) AD(506)	99,320	AD(2,303)	9
Li,2017 (17)	AD	Sweden	55.6‡	1964-2010	NA	CD:1.20 (0.93-1.51)* UC:1.03 (0.85-1.25)*	SIR	NA	AD(70) AD(110)	NA	NA	8
Sand,2022 (18)	AD	Denmark	CD:36 (23-56)§ UC:43 (29-62)§	1977-2018	13.54years	CD:0.91 (0.76-1.09) UC:1.10 (1.01-1.19)	HR	IBD(88,985) CD(27,090) UC(61,895)	AD(757) CD(131) UC(626)	884,108	AD(8,813)	9
Vadstrup,2020 (19)	AD	Danmark	> 0	2003-2016	NA	CD:1.05 (0.57-1.94) * UC:1.08 (0.79-1.49) *	OR	CD(10,302) UC(22,144)	AD(0.2%) AD(0.4%)	32,446	AD (0.2%) AD(0.3%)	8
Zhang,2021 (20)	AD	Taiwan,China	60.64 ± 10.75†	1998-2011	16years	IBD:6.19 (3.31-11.57) CD:7.53 (2.67-21.28) UC:6.77 (2.82-16.22)	HR	IBD(1,742) CD(584) UC(1,158)	AD(33) AD(11) AD(22)	17,420	AD(32)	9
Turner,2013 (21)	ALS	UK	> 0	1999-2011	NA	CD:1.22 (0.91-1.59) UC:1.22 (1.00-1.48)	RR	NA	ALS(54) ALS(106)	NA	ALS exp(44.5) ALS exp(87.2)	7
Bernstein,2021 (22)	Dementia	Canada	36 (25-52)§	1984-2018	NA	IBD:1.18 (1.02-1.36)* CD:1.44 (1.16-1.78)* UC:1.01 (0.85-1.19)*	HR	IBD(9,247) CD(4,253) UC(4,994)	NA	85,691	NA	9
Dregan,2015 (23)	Dementia	UK	CD:42 ± 18† UC:47 ± 18†	2002-2013	NA	CD:1.55 (1.06-2.26) UC:1.23 (0.92-1.64)	HR	CD(7,705) UC(12,335)	Dementia(47) Dementia(93)	308,843	Dementia (2,805)	9
Li,2017 (17)	Dementia	Sweden	55.6‡	1964-2010	NA	CD:1.17 (1.02-1.35)* UC:1.24 (1.13-1.37)*	SIR	NA	Dementia(203) Dementia(407)	14,411,278	Dementia (236,278)	8
Sand,2022 (18)	Dementia	Danmark	CD:36 (23-56)§ UC:43 (29-62)§	1977-2018	13.54years	CD:1.15 (1.05-1.27) UC:1.07 (1.01-1.12)	HR	IBD(88,985) CD(27,090) UC(61,895)	Dementia(2,076)Dementia(455) Dementia(1,621)	884,108	Dementia (23,011)	9
Sun,2021 (24)	Dementia	UK	CD:56.9 ± 8.1† UC:58.1 ± 7.8†	2006-2010	11.58years	IBD:1.14 (0.94-1.39) CD:1.20 (0.84-1.71) UC:1.12 (0.89-1.42)	HR	IBD(5,578) CD(1,826) UC(3,952)	Dementia(100) Dementia(NA) Dementia(NA)	491,997	Dementia (6,709)	9
Zhang,2021 (20)	Dementia	Taiwan,China	60.64 ± 10.75†	1998-2011	16years	IBD:2.54 (1.91-3.37) CD:2.29 (1.42-3.69) UC:2.69 (1.89-3.85)	HR	IBD(1,742) CD(584) UC(1,158)	Dementia(95) Dementia(30) Dementia(65)	17,420	Dementia(250)	9
Zingela,2021 (25)	Dementia	Gemany	70.9 ± 7.3†	1995-2014	15years	IBD:1.22 (1.07-1.39)* CD:1.17 (0.93-1.47)* UC:1.25 (1.07-1.46)*	HR	IBD(3,850) CD(1,431) UC(2,419)	Dementia(26.3%) Dementia(NA) Dementia(28.1%)	3,850	Dementia (23.8%)	9
Avasarala,2021 (26)	MS	USA	49.4 ± 16.75†	2010-2018	NA	IBD:1.32 (1.03-1.71)	IRR	IBD(208,681)	MS(80)	827,045	MS(235)	8
Burisch,2019 (27)	MS	Denmark	45.8(33.8–60.7)§	2007-2016	5.4 (2.9–7.8)¶	IBD:1.72 (1.05-2.83) CD:1.76 (0.70-4.48) UC:1.58 (0.83-3.02) IBD-U:2.50 (0.63-9.99)	IRR	IBD(14,377) CD(3,879) UC(9,212) IBD-U(1,286)	MS(21) MS(6) MS(12) MS(3)	71,885	NA	8
Card,2016 (28)	MS	UK	47.2‡	1987-2011	NA	IBD:2.82(2.27-3.50)	OR	IBD(56,097) CD(18,204) UC(27,108)	MS(265) MS(89) MS(128)	280,382	MS(913)	8
Gupta,2005 (29)	MS	UK	CD:(42.7 ± 18.4)† UC:(48.9 ± 17.8)†	1988-1997	CD:4.8 (1.9–6.5)¶ UC:5.0 (2.3–6.7)¶	CD:2.12 (0.94-4.50)* UC:2.63 (1.29-5.15)*	IRR	CD(7,988) UC(12,185)	MS(11) MS(15)	80,666	MS(50)	9
Park,2019 (30)	MS	Korea	41.7 ± 16.4†	2012-2016	NA	IBD:2.89 (1.02-8.42)* CD:10.73 (1.11-103.8)* UC:1.60 (0.39-6.61)*	HR	IBD(35,581) CD(11,803) UC(23,737)	MS(6) MS(3) MS(3)	142,324	NA	8
Villumsen,2019 (31)	MSA	Denmark	≥15	1977-2014	9.4years	IBD: 1.41 (0.82-2.44)	HR	IBD(76,477)	MSA(13)	7,548,259	MSA(866)	9
Coates,2021 (32)	PD	USA	45.5 ± 10.3†	2005-2014	3.8years	IBD:1.01 (0.72-1.42) CD:1.33 (0.80-2.21) UC:0.81 (0.51-1.29)	HR	IBD(154,051) CD(NA) UC(NA)	PD(68) PD(35) PD(33)	154,051	PD(64)	8
Kim,2022 (16)	PD	Korea	55.4 ± 11.0†	2009-2017	6years	IBD:1.56 (1.24-1.97) CD:1.03 (0.58-1.84) UC:1.69 (1.32-2.15)	HR	IBD(24,830) CD(4,454) UC(20,376)	PD(98) PD(12) PD(86)	99,320	PD(256)	9
Li,2012 (33)	PD	Sweden	> 0	1964-2007	NA	CD:0.62 (0.33-1.07) UC:1.23 (0.90-1.64)	SIR	CD(22,750) UC(27,881)	PD(13) PD(46)	NA	NA	8
Lin,2016 (34)	PD	Taiwan,China	46.9 ± 17.0†	2000-2011	7.3years	IBD:1.35 (1.08-1.68) CD:1.40 (1.11-1.77) UC:0.94 (0.49-1.84)	HR	IBD(8,373) CD(NA) UC(NA)	PD(106) PD(97) PD(9)	33,492	PD(290)	9
Loosen,2023 (35)	PD	Germany	59.7 ± 12.8†	2005-2020	6.2years	CD:1.23 (0.90-1.69)* UC:0.96 (0.75-1.23)*	HR	CD(7,544) UC(10,450)	PD(2.1%) PD(2.1%)	17,944	PD(1.8%) PD(2.1%)	9
Park,2019 (36)	PD	Korea	39.91 ± 16.62†	2010-2013	4.9years	IBD:1.87 (1.43-2.44) CD:2.23 (1.12-4.45) UC:1.85 (1.38-2.48)	HR	IBD(38,861) CD(12,631) UC(26,230)	PD(92) PD(15) PD(77)	116,583	PD(134)	7
Peter,2018 (37)	PD	USA	50.8 ± 16.8†	2000-2016	NA	IBD:1.28 (1.14-1.44) CD:1.26 (1.03-1.53) UC:1.31 (1.14-1.51)	IRR	IBD(144,018) CD(56,507) UC(84,436)	PD(371) PD(122) PD(243)	720,090	PD(1,425)	9
Vadstrup,2020 (19)	PD	Denmark	> 0	2003–2016	NA	CD:1.24 (0.73-2.10)* UC:1.58 (1.18-2.12)*	OR	CD(10,302) UC(22,144)	PD(0.3%) PD(0.5%)	NA	PD(0.2%) PD(0.3%)	8
Villumsen,2019 (31)	PD	Denmark	≥15	1977-2014	9.4years	IBD: 1.22 (1.09-1.35)	HR	IBD(76,477)	PD(335)	7,548,259	PD(39,784)	9
Weimers,2019 (38)	PD	Sweden	45 ± 18†	2002-2014	6.3years	IBD:0.9(0.7-1.1) CD:0.9 (0.6-1.4) UC:0.8 (0.6-1.1) IBD-U:1.0(0.5-1.9)	HR	IBD(39,652) CD(11,418) UC(24,422) IBD-U(3,812)	PD(103) PD(23) PD(69) PD(11)	396,520	PD(839)	9

AD, Alzheimer’s disease; ALS, amyotrophic lateral sclerosis; MS, multiple sclerosis; MSA, multiple system atrophy; PD, Parkinson’s disease; IBD, inflammatory bowel disease; IBD-U, IBD-unclassified; CD, Crohn’s disease; UC, ulcerative colitis; OR:odds ratio; HR, hazard ratio; SIR, standardized risk ratio; RR, rate ratio; IRR, incidence rate ratio.

^†^ mean ± SD ^‡^ mean ^§^ median (Q1-Q3) ^¶^ years follow-up, median (Q1-Q3) * unadjusted effect estimate.

Diagnosis of IBD and neurodegenerative diseases was built on related medical records or diagnostic codes found in medical record database systems or commercial claims databases, along the lines of the International Classification of Diseases codes. Based upon the NOS, almost all original studies acquired at least eight stars, with only two studies receiving seven stars, indicating a minuscule overall risk of design bias. (**Supplementary Table 2**).

### 3.2 The extra risk of AD in IBD patients

**Figure 1:**
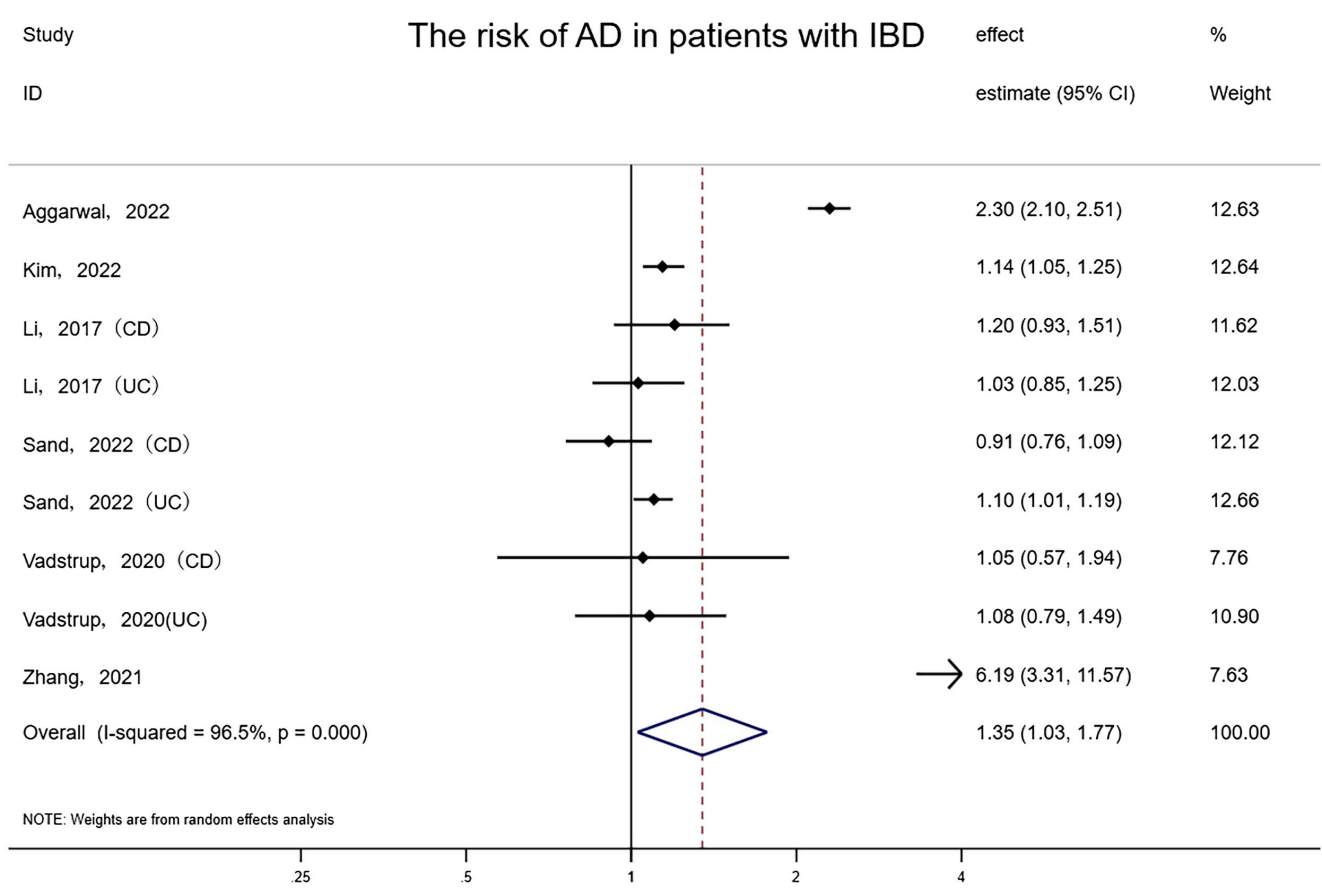
Forest plot of the associations between inflammatory bowel diseases and Alzheimer’s disease development.

Overall, 6 studies involving at least 66,381,877 subjects (one study did not report how many patients were analyzed) indicated a meaningful correlation between IBD and AD (random-effects HR= 1.35, 95% CI: 1.03–1.77, P=0.031), with nonnegligible heterogeneity (I²= 96.5%) (**Figure 1**). To evaluate the consistency of the pooled results, an influence analysis strategy was performed that excluded each of the incorporated studies individually, in order to determine the likelihood of any excessive impact by them. Interestingly, after eliminating the study implemented by Zhang et al, 2021, the statistical significance was submerged (random-effects HR= 1.19, 95% CI: 0.91–1.56). We reevaluated the study of Zhang et al., believing that the reason for this situation is that the study population in Zhang et al. ’s study was the oldest among all studies (the mean age was 60.64), and the follow-up time (16 years) was longer than other studies, which may lead to a substantially elevated prevalence of AD than other studies. The results of the combined effect were reliable and we should not exclude the study of Zhang et al. because of its high quality. The funnel plot revealed that there was no asymmetry, and no evidence of small-scale study effects or publication bias was detected upon evaluation by Egger’s test (P = 0.914).

### 3.3 The extra risk of Dementia in IBD patients

**Figure 2:**
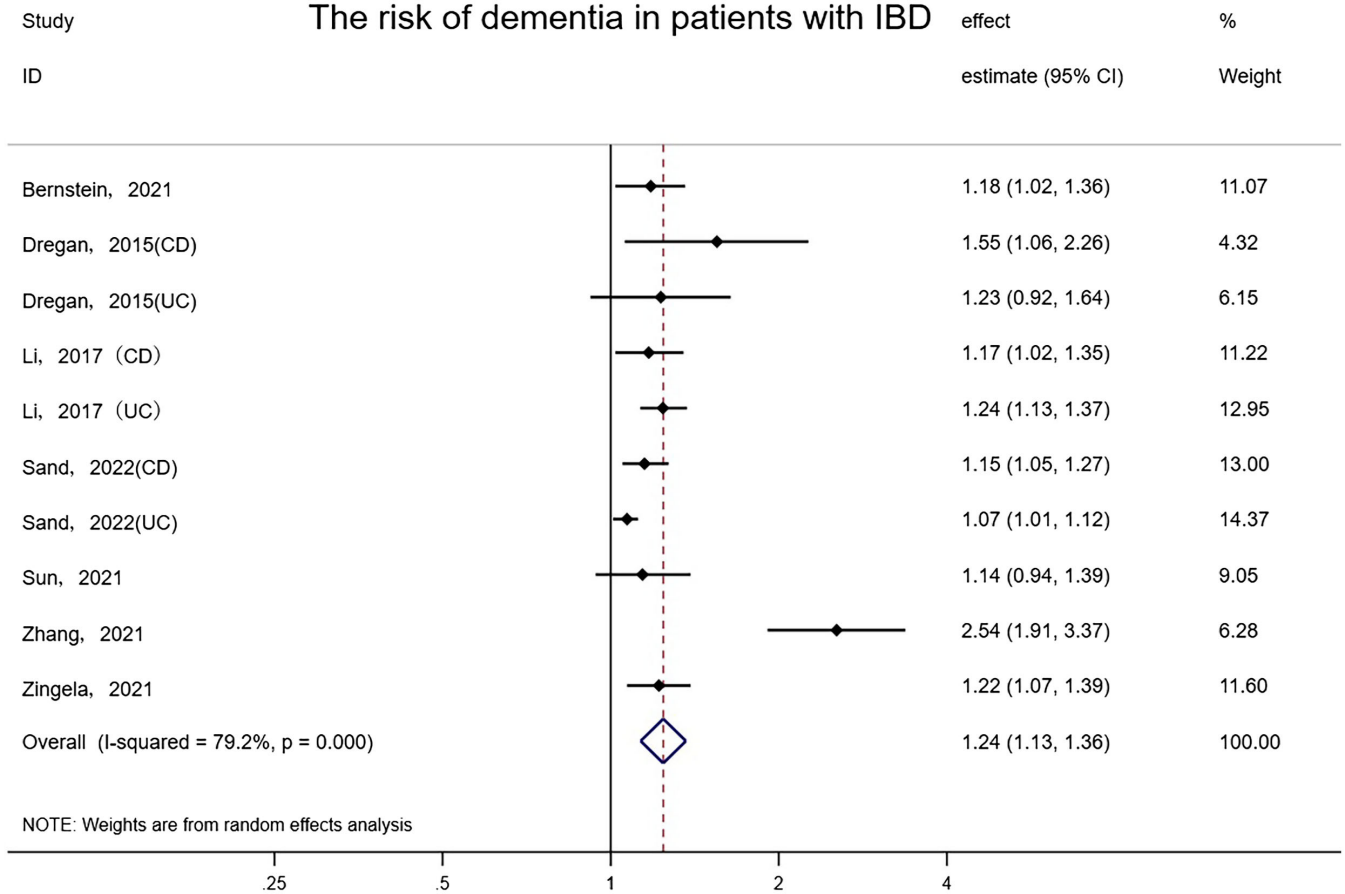
Forest plot of the associations between inflammatory bowel diseases and dementia development.

Seven studies involving approximately 16,332,899 subjects (one study did not report the number of patients with IBD) reported the risk of dementia in patients with IBD and controls. People with IBD had a notable increase in the occurrence of dementia compared to controls (random-effects HR = 1.24, 95% CI: 1.13–1.36, I²= 79.2%, P<0.001) (**Figure 2**). The effect estimates remained stable and consistent after eliminating any single study according to the results of sensitivity analyses. Also, we found there may be latent publication bias (Egger’s test P=0.019). We further assessed the summarized effect through the trim-and-fill method (42). The amended trim-and-fill method assigned 6 hypothetically missing studies to attenuate the risk. After 6 hypothesized missing studies were imputed via the trim and fill procedure, the correlation between IBD and dementia remained (Random-effects:1.107, 95% CI: 1.011–1.211, P=0.028, Q=96.909, P<0.001) (**Supplementary Figure 2**).

### 3.4 The extra risk of MS in IBD patients

**Figure 3:**
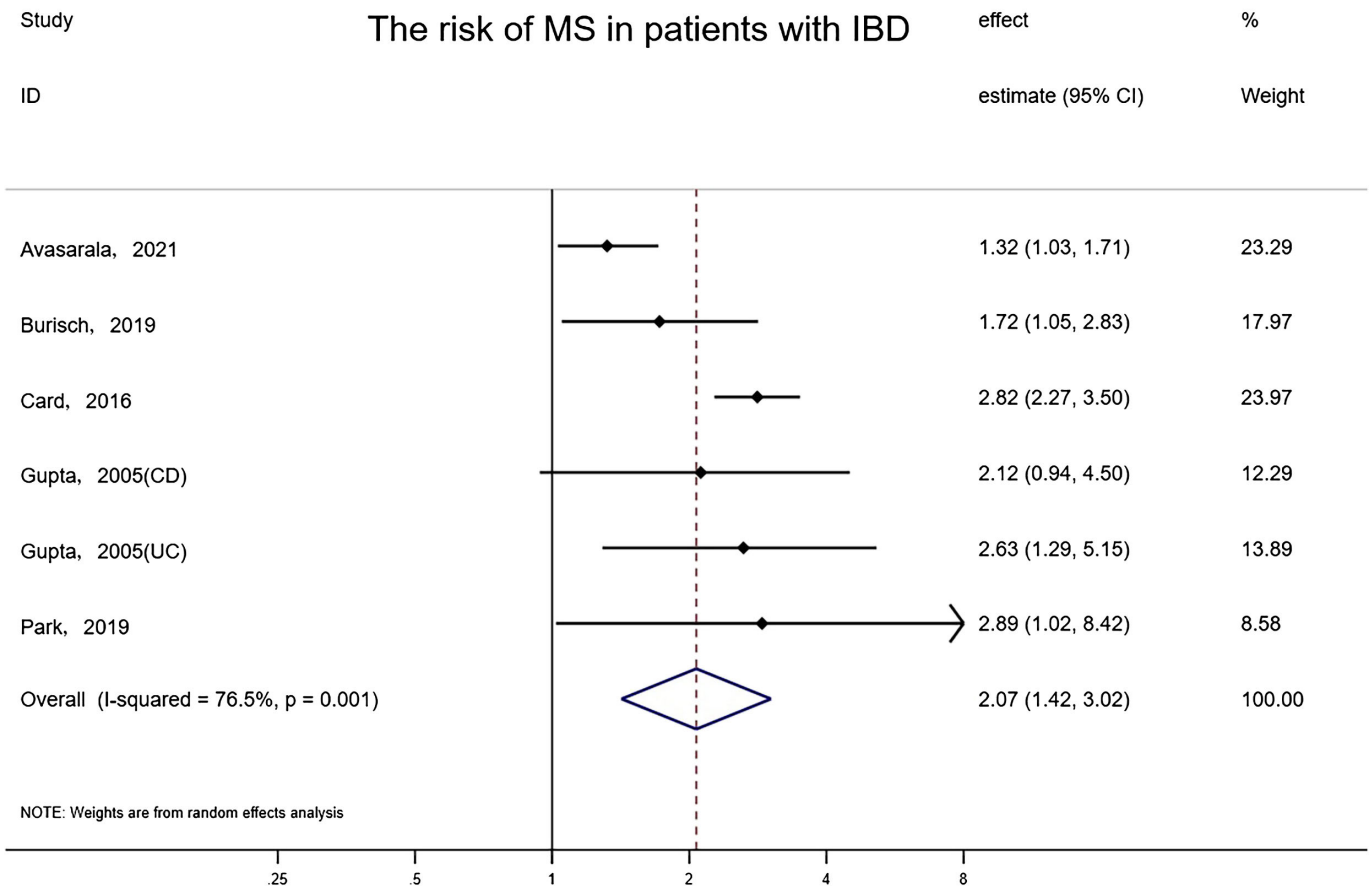
Forest plot of the associations between inflammatory bowel diseases and multiple sclerosis development.

The overall risk of MS in relation to IBD among five studies involving 1,737,211 subjects reporting exposure and outcome events, HR was markedly higher than that in IBD-free populations (random-effects HR=2.07, 95% CI:1.42–3.02, I²=76.5%, P<0.001) (**Figure 3**). Sensitivity analyses indicated that the effect was stable and consistent when any single study was eliminated. The funnel plot revealed that there was no asymmetry, and no publication bias or small-scale study effects were detected by Egger’s test (P=0.963).

### 3.5 The extra risk of PD in IBD patients

**Figure 4:**
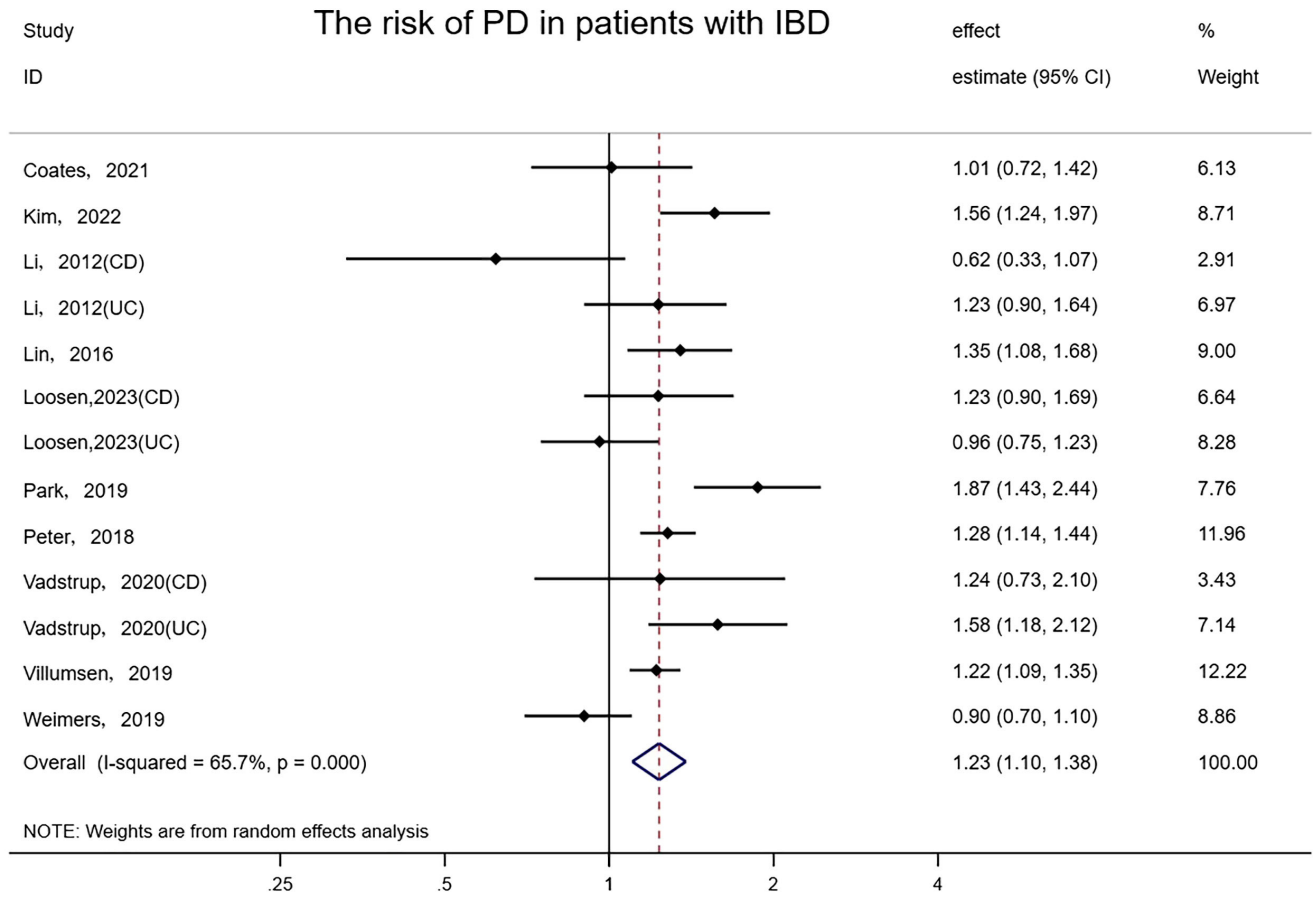
Forest plot of the associations between inflammatory bowel diseases and Parkinson’s development.

Pooled results from 10 studies involving 9,673,542 subjects (two original studies did not provide the number of Non-IBD controls) suggested there was a meaningful association between IBD and PD (random-effects HR = 1.23, 95%CI: 1.10–1.38, I² = 65.7%, P<0.001) (**Figure 4**). Excluding one study that received 7 stars for NOS (Park, 2019), the risk association for PD in IBD patients remained and the heterogeneity was slightly reduced (random-effects HR = 1.20, 95%CI: 1.08–1.33, I²= 57.1%, P=0.01). The elimination of any study did not affect the consistency and stability of the effect. No evidence of asymmetry was found in the funnel plot, and Egger’s test detected no small-scale study effects or publication bias (P = 0.678).

### 3.6 The extra risk of IBD in patients with neurodegenerative diseases

**Figure 5:**
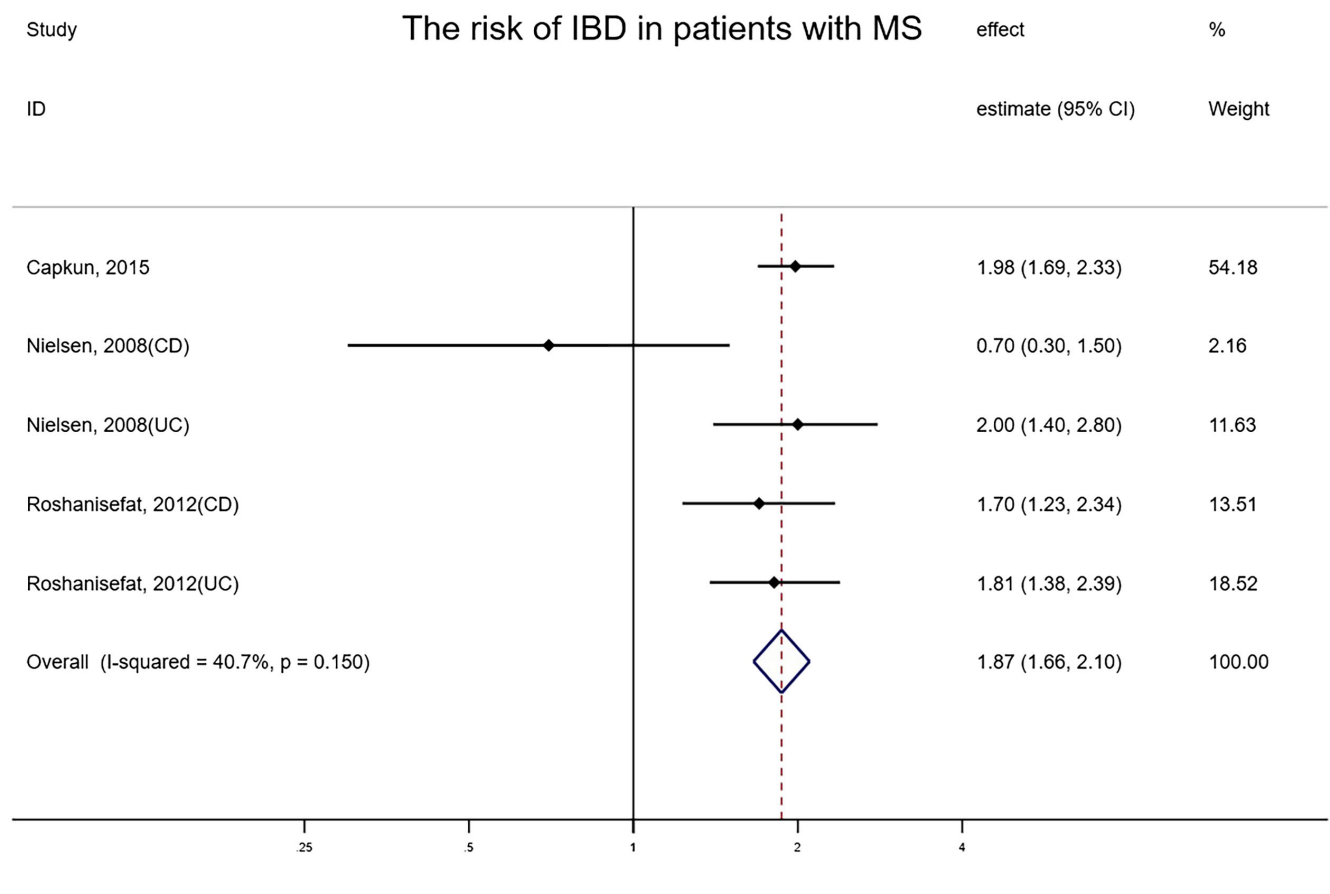
Forest plot of the risk of IBD in patients with neurodegenerative diseases.

Only three studies explored the risk of IBD in patients with neurodegenerative diseases, with all types of neurodegenerative diseases being MS (39–41). The overall risk of IBD among three studies involving 330,734 subjects reporting exposure and outcome events, HR was remarkably higher than that in general populations (fixed-effects HR=1.87, 95% CI:1.66–2.10, I² = 40.7%, P<0.001) (**Figure 5**). When any single study was excluded, the effect estimates remained consistent and stable. The funnel plot illustrated no asymmetry and Egger’s test detected no small-study effects or publication bias (P = 0.075).

### 3.7 Subgroup analyses

On account of the small number of studies, we did not conduct a subgroup analysis of the risk outcome of IBD in patients with neurodegenerative diseases. We performed a subgroup analysis of the results of the risk analysis for neurodegenerative diseases in patients with IBD based on age, the types of IBD, study country and covariates adjustment to identify potential sources of heterogeneity within the eligible studies. (**Table 2**).

**Table 2:** Subgroup analyses—association between IBD and neurodegenerative diseases, stratified by age, IBD subgroup, study country and covariate adjustment.

Age	Effect estimate (95%CI)	IBD subgroup	Effect estimate (95%CI)	Study country	Effect estimate (95%CI)	Covariate adjustment	Effect estimate (95%CI)
AD≥50 (16, 17, 20)	1.44 (1.04-2.00)	AD-CD (15–20)	1.69 (0.87-3.28)	AD-Europe (17–19)	1.07 (1.00-1.14)	AD-adjusted (15, 16, 18, 20)	1.59 (1.08-2.34)
				AD-America (15)	2.30 (2.10-2.51)		
AD<50 (18)	1.02 (0.85-1.22)	AD-UC (15–20)	1.52 (0.99-2.32)	AD-Asia (16, 20)	2.58 (0.49-13.52)	AD-unadjusted (17, 19)	1.09 (0.95-1.24)
Dementia≥50 (17, 20, 24, 25)	1.34 (1.11-1.62)	Dementia-CD (17, 18, 20, 22–25)	1.27 (1.13-1.43)	Dementia-Europe (17, 18, 23–25)	1.16 (1.10-1.24)	Dementia-adjusted (18, 20, 23, 24)	1.32 (1.11-1.56)
				Dementia-America (22)	1.18 (1.02-1.36)		
Dementia<50 (18, 22, 23)	1.14 (1.06-1.22)	Dementia-UC (17, 18, 20, 22–25)	1.25 (1.14-1.36)	Dementia-Asia (20)	2.54 (1.91-3.37)	Dementia-unadjusted (17, 22, 25)	1.21 (1.14-1.29)
MS≥50	—	MS-CD (27, 29, 30)	2.19 (1.21-1.83)	MS-Eupore (27–29, 31)	2.58 (2.14-3.10)	MS-adjusted (26–28)	1.87 (1.09-3.23)
				MS-America (26)	1.32 (1.02-1.70)		
MS<50 (26–30)	2.07 (1.42-3.02)	MS-UC (27, 29, 30)	1.96 (1.25-3.06)	MS-Asia (30)	2.89 (1.01-8.30)	MS-unadjusted (29, 30)	2.48 (1.56-3.95)
PD≥50 (16, 35, 37)	1.25 (1.05-1.49)	PD-CD (16, 19, 32–38)	1.21 (1.03-1.41)	PD-Europe (19, 31, 33, 35, 38)	1.12 (0.97-1.30)	PD-adjusted (16, 31–34, 36–38)	1.24 (1.08-1.42)
				PD-America (32, 37)	1.20 (0.98-1.48)		
PD<50 (32, 34, 36, 38)	1.23 (0.89-1.70)	PD-UC (16, 19, 32–38)	1.23 (1.02-1.48)	PD-Asia (16, 34, 36)	1.56 (1.30-1.87)	PD-unadjusted (19, 35)	1.22 (0.96-1.55)

#### 3.7.1 Age-specific analysis

Out of the 6 studies on AD and 10 studies on dementia, we found AD and dementia incidence was greater among older patients with IBD (age≥50) than among younger patients with IBD (age<50). For AD, the integrated HR for older IBD patients was 1.44 (95%CI: 1.04–2.00, P=0.30) in four studies (Kim 2022, Li 2017-CD, Li 2017-UC, Zhang 2021), while for younger patients the pooled HR was 1.02 (95% CI: 0.85–1.22, P=0.845) in two studies (Sand 2022-CD, Sand 2022-UC). For dementia, the pooled effect estimate for older IBD patients was 1.34 (95% CI: 1.11–1.62, P = 0.002) in five studies (Li 2017-CD, Li 2017-UC, Sun 2021, Zhang 2021, Zingela 2021), and for younger patients was 1.14 (95% CI: 1.05–1.22, P = 0.001) in five studies (Bernstein 2021, Dregan 2015-CD, Dregan 2015-UC, Sand 2022-CD, Sand 2022-UC). Based on our analysis, in comparison with younger patients, elderly individuals with IBD exhibited a slightly elevated risk of AD and dementia compared with IBD-free controls. There did not appear to be any substantial dissimilarity in the incidence of Parkinson’s disease among IBD patients in different age groups. Regrettably, insufficient studies have been carried out regarding the potential correlation between MS and elderly IBD patients, we did not have enough evidence to test whether elderly IBD patients are at an elevated risk of MS in comparison to younger patients (**Supplementary Table 3**).

#### 3.7.2 IBD subtype analysis

For our synthetic analyses of IBD and four neurodegenerative diseases, the risk of AD and MS in CD patients was marginally greater than in UC patients, while the risk of dementia and PD did not differ markedly among the distinct IBD subgroups (**Supplementary Table 4**).

#### 3.7.3 Study location analysis

IBD patients in Asia appeared to have an obviously increased risk of neurodegenerative diseases than patients in Europe and America, regardless of any of the four diseases. For dementia, the morbidity risk in IBD patients in Asia was nearly twice as high as that in IBD patients in America and Europe. Asian patients with IBD have a greater than twofold risk of developing AD compared to European IBD patients and more than double the risk of MS compared to American IBD patients. But the results of our subgroup analysis were limited by the amount of studies conducted in Asia. No significant difference seemed to exist between European and American populations in the risk of developing dementia and PD. However, for AD, IBD patients in America are at approximately twice the risk of IBD patients in Europe, but this result is based on the limited number of studies on IBD patients in America (only one study). While for MS, the risk of developing it in patients with IBD in Europe was approximately double that in IBD patients in America. However, this conclusion was also limited by the small number of studies (only one study examined the risk of developing MS in IBD patients in America) (**Supplementary Table 5**).

#### 3.7.4 Covariate adjustment analysis

The risk of AD and dementia among IBD patients rose after being adjusted for age, gender, comorbidibility, and other factors, while for MS, the risk of morbidity risk among IBD patients decreased after adjustment. The risk of PD occurrence among IBD patients did not differ significantly after adjusting for covariates. (**Supplementary Table 6**).

## 4 Discussion

This meta-analysis included 27 higher-quality longitudinal studies, 24 of which investigated the risk of various neurodegenerative diseases in patients with IBD. The study focused on systematic analyses of the correlation between IBD and four main neurodegenerative diseases (AD, dementia, MS and PD). We found that IBD is in connection with a higher incidence of the above four neurodegenerative diseases. Additionally, one study investigated the risk of amyotrophic lateral sclerosis (ALS) among IBD patients, and another examined multiple system atrophy (MSA). Although both articles reported an increased incidence of ALS or MSA among IBD patients in comparison with the general population, confined by the number of studies, we were unable to conduct a systematic review of these two neurodegenerative diseases. However, it was suggested that IBD may be associated with a wider range of neurodegenerative diseases, beyond the four that were the focus of our study. we also identified three cohort studies that explored the risk of IBD among MS patients (39–41). A pooled analysis of these studies determined that MS patients have a higher risk of developing IBD compared to those without IBD. This also verified the two-directional correlation of the brain-gut axis. Intestinal inflammation not only increases the risk of neurodegeneration, but neurodegeneration also leads to the occurrence of intestinal inflammation. However, it has not been thoroughly studied whether other neurodegenerative diseases cause IBD. More high-quality cohort studies are needed to verify this two-directional brain-gut association.

Several previous meta-analyses initially examined the risk of developing various neurodegenerative diseases among patients with IBD. However, it should be noted that studies included in the pooled analysis in previous meta-analyses were not restricted to specific study types and may have included cross-sectional and case-control studies, which may introduce potential bias. Fu et al. conducted a meta-analysis that summarized the risk association between different gastrointestinal diseases, including IBD, and PD and AD (7). Although the study explored a wide range of gastrointestinal diseases, there were limited studies that primarily investigated IBD and its relationship with these two neurodegenerative diseases. To explore this link, the authors aggregated data from four studies relevant to IBD and PD and reviewed one study on IBD and AD. Wang et al. conducted a thorough meta-analysis investigating the bidirectional association between IBD and MS (8). The analysis of 17 studies revealed that patients with MS had a greater prevalence of IBD than controls (RR = 1.53). Patients with IBD had a greater prevalence of MS than controls (RR = 1.91). Nevertheless, Wang et al. did not restrict the type of studies considered, and among them were six studies of moderate quality, which could pose potential bias risks. Zhu et al. analyzed 9 studies investigating the risk of PD among IBD patients, which included 6 retrospective cohort studies (9). They clearly demonstrate that the risk of PD is significantly higher in IBD patients than in the general population (aHR = 1.24). Zuin et al. summarized three studies to determine the risk of AD in individuals with IBD (10). The available data backs up the claim that IBD patients have a 1.52-fold elevated risk of developing AD in comparison to the general population. Our meta-analysis has extended the scope of the research, incorporating a higher number of studies than previous meta-analyses. We have limited the selection of studies to longitudinal ones, in order to decrease the possible risks of bias.

There was considerable heterogeneity in this meta-analysis, possibly reflecting the diversity of study characteristics included (such as age of the study population, whether environmental factors were adjusted, duration of follow-up, different diagnostic criteria for the disease or different treatment of IBD, etc.). Accordingly, a fixed-effects or random-effects model was selected based on different levels of heterogeneity while subgroup analyses were performed to identify potential sources of heterogeneity. Sensitivity analyses were used to ensure the stability and reliability of our results, and no significant publication bias was found in this study.

Aging is the major risk factor for neurodegeneration (43). Our analysis indicates that older patients with IBD are at a higher risk of developing AD and dementia compared to younger patients with IBD. This is consistent with the theory that cellular senescence increases susceptibility to AD (44–47). Neuroinflammation and the accumulation of amyloid β-protein are more likely to occur in older patients (43). However, the subgroup analysis according to age did not indicate that older patients with IBD were at a higher risk of developing PD compared to younger patients with IBD. Furthermore, we do not have information on the risk of developing MS in older patients with IBD, and therefore cannot compare the risk of developing MS in IBD patients of different ages.

The two subtypes of IBD, CD and UC, differ in terms of lesion location and extension, and have distinct phenotypic spectrums (3). Thus, subgroup analyses were performed for different types of IBD to investigate whether CD and UC have varying effects on the incidence of neurodegenerative diseases. It was found that the risk of AD and MS was higher in CD patients than in UC patients. It seems that CD is more closely associated with an increased risk of AD and MS. However, the underlying reasons for this association remain unclear and require further data and theory to verify.

Environmental factors such as diet, sleep patterns, physical activity and chronic lifestyle stress are thought to be closely related to the development of neurodegenerative diseases (48). Environmental factors, including urbanization, pollution, and diet, are also believed to be significant contributors to the development of IBD (49). Several environmental risk factors are commonly associated with the pathogenesis of both neurodegenerative diseases and IBD, which could potentially confound the association. To investigate potential sources of heterogeneity, we conducted subgroup analyses that adjusted for country/region and covariates (yes, no). Our findings indicate that Asian patients with IBD are at a higher risk of developing neurodegenerative diseases than their European and American counterparts, regardless of which of the four neurodegenerative diseases. For AD, American IBD patients had approximately twice the risk of developing MS compared with European IBD patients, whereas for MS, European IBD patients had approximately twice the risk of developing MS compared with American IBD patients. This suggests a potential difference in genetic or environmental factors that may contribute to the development of neurodegenerative diseases in Asian populations. However, the results of these subgroup analyses are limited by the number of studies, and more studies are still needed to further explore the risk of IBD patients in different regions. Among the 24 included studies exploring the risk of neurodegenerative diseases in IBD patients, a total of 18 studies had adjusted confounding factors to some extent. The association of increased risk of four neurodegenerative diseases in IBD patients remained regardless of adjustment for confounding factors. However, after adjustment for age, gender, comorbidities and other factors, the risk of AD and dementia increased in IBD patients, and the risk of MS decreased in IBD patients. There was no significant difference in the risk of PD in IBD patients before and after adjustment. Environmental factors in different countries or regions appear to be a significant source of study heterogeneity. However, subgroup analysis on a single environmental confounder may be challenging due to the accounting of various confounding factors in each study.

The potential mechanisms underlying the relationship between IBD and neurodegenerative diseases are complex and multifactorial. First, intestinal inflammation damages the intestinal mucosal barrier, allowing bacteria or bacterial by-products to enter the bloodstream and activate immune cells (50). Lipopolysaccharide (LPS) and activated cytokines can penetrate the blood-brain barrier to prompt the inflammatory reaction of the central nervous system, leading to the degeneration of certain neurons (51). Second, the link between IBD and neurodegeneration may be mediated by alterations in the gut microbiota. Disruption of microbial diversity in the gastrointestinal duct of IBD patients could potentially result in a reduction in anti-inflammatory metabolites and an elevation in neurotoxic metabolites (52, 53). Inflammatory biopolymers, such as LPS and enterotoxins, specifically damage the intestinal wall, allowing neurotoxic metabolites to move from the gastrointestinal tract into the circulatory and central nervous systems (53–55). The microbiota also plays a crucial role in the development of IBD (56). Neurodegenerative diseases may cause disorders in the intestinal flora. Patients with AD, ALS, and PD exhibit marked changes in the structure of gut microbiota, which display evident proinflammatory characteristics (57–60). Third, genetic overlap may explain the link. Several variants in independent genomic loci have been found to associate with both PD and IBD (61). LRRK2 genetic mutations are the most established genetic predisposing factor for PD, and also potentially enhance the likelihood of CD (62, 63). Three single nucleotide polymorphisms have been identified to have a crucial association with both IBD and MS. Individuals carrying the allele in the -1082 G/A polymorphism of the interleukin-10 gene are at a higher risk of developing both UC and AD (64). Besides, prolonged inflammation of the gut can lead to the accumulation of certain abnormal substances. Chronic gastrointestinal inflammation may cause the buildup of α-synuclein and β-amyloid plaque. The transportation of these substances to the central nervous system occurs through the vagus nerve (51, 53, 65). Additionally, individuals with IBD may be more susceptible to experiencing blood clotting events due to disease activity, corticosteroid use, hospitalization, or surgery (66–68). Thrombotic events may increase the risk of developing vascular dementia. In addition to alterations in gut microbiota and genetic overlap, changes in neurotransmitters used by both the enteric nervous system (ENS) and the central nervous system (CNS) leading to ENS dysfunction can also explain the development of IBD caused by neurodegenerative diseases (69, 70). The ENS is a reflex control system that extensively regulates digestive function, collaborating with the CNS. The ENS and CNS share functional and chemical similarities, as both systems utilize specific neurotransmitters (69). Dysregulation of neurotransmitters occurs in numerous neurodegenerative conditions, such as the significant loss of cholinergic neurons in AD and disruptions in DA metabolism in PD (71, 72). GABA also holds significance in the pathogenesis of AD, PD and MS (73, 74). An abnormality in glutamate metabolism is observed in AD patients, and there is substantial evidence indicating the vital role of glutamate in the onset and progression of IBD (74, 75).

Several included studies have demonstrated that administering whichever of 5-aminosalicylic acid, thiopurines, glucocorticoids, biologic agents, and anti-TNF therapy has a protective effect against the development of neurodegenerative diseases in patients with IBD, ranging from AD, PD, and dementia (15, 19, 23, 26, 30, 36–38). These findings provide evidence for the correlation between the brain-gut axis, which suggests that managing IBD inflammation may lower the incidence of neurodegenerative diseases. However, it is worth noting that Aggravel et al. demonstrated that while the utilization of immunomodulators, other biologic agents and TNF-α agents can reduce the risk of AD, the use of prednisone in IBD patients raised the risk of AD (15). Furthermore, Avasarala et al. discovered an escalated risk of MS for IBD patients after anti-TNF-α exposure, particularly for CD patients (26). Aside from the impact of anti-TNF-α therapy on definite MS, there is a connection between anti-TNF-α agents and newly onset of MS. It is uncertain whether these demyelinating episodes are coincidental or causally linked with the usage of TNF-α blockers (76). Nonetheless, vigilance is necessary despite the absence of a clear association.

In this study, some limitations must be noted. First, it is not allowed to demonstrate a causal relationship as the eligible studies were observational in design. Second, some potential confounding factors cannot be ruled out although a great number of the qualified studies adjusted the results for age, gender, comorbidity and other factors. Meanwhile, it was not feasible to integrate models from studies that made adjustments for the identical set of potential confounders. Third, despite a random-effects model being implemented in most cases, it is important to approach some of the outcomes of this meta-analysis with care due to the relatively high heterogeneity that was evident in the overall primary analysis. Fourth, this systematic review included studies from countries in Europe, North America, and Asia (two countries), but there are also many parts of the world (e.g., Latin America, the Middle East, Asia and other countries in Asia) where the incidence of IBD has been increasing and for which no studies were included in this review. It is unclear whether findings from this study would be generalizable to IBD patients from these other regions. Fifth, IBD is usually diagnosed among younger individuals whereas neurodegenerative diseases tend to be more frequently diagnosed in older individuals. However, due to the limited availability of data, age was only allocated into merely two subgroups (<50 and ≥50 years). Further verification is required concerning the risk factors of various neurodegenerative diseases among older people with IBD. Furthermore, the studies included in the meta-analysis were deemed acceptable in terms of quality, indicating a moderate to low probability of bias built on the NOS criteria. Nonetheless, to analyze the heterogeneity causes, it would be necessary to collect individual participant information from extensive cohort studies. Finally, the identification of diseases through diagnostic codes is subject to unavoidable underdiagnosis and misclassification.

Despite the above limitations, our study has critical advantages. This meta-analysis includes data from large longitudinal studies from Europe, America and Asia, which are likely to accurately reflect common patients with IBD in clinical practice. We also searched for relevant literature from previous meta-analyses. As a result, we believe that it is unlikely that we have missed any published trials due to our comprehensive search.

In conclusion, this meta-analysis of longitudinal studies demonstrates a substantial association between IBD and an elevated risk of incident neurodegeneration. However, to confirm the exact association, more high-quality prospective longitudinal studies are imperative. Clinicians should be alert to regard the increased risk of neurodegeneration and IBD comorbidity. It seems necessary to carry out regular neurological screening for IBD patients to detect and intervene in neurodegenerative changes early during clinical practice.

## Data availability statement

The original contributions presented in the study are included in the article/**Supplementary Material**. Further inquiries can be directed to the corresponding authors.

## Author contributions

JZ: Formal analysis, Writing – original draft. YY: Writing – original draft, Data curation. HW: Data curation, Writing – review & editing. HZ: Data curation, Writing – original draft. XRY: Formal analysis, Methodology, Supervision, Writing – review & editing. XYY: Conceptualization, Supervision, Writing – review & editing.

## References

[1] Alatab S Sepanlou SG Ikuta K Vahedi H Bisignano C Safiri S . The global, regional, and national burden of inflammatory bowel disease in 195 countries and territories, 1990-2017: a systematic analysis for the global burden of disease study 2017. Lancet Gastroenterol Hepatol. (2020) 5(1):17–30. doi: 10.1016/s2468-1253(19)30333-4 31648971 PMC7026709

[2] Prince M Wimo, A Guerchet M Ali G Wu Y Prina M . The global impact of dementia: an analysis of prevalence, incidence, cost and trends . Alzheimer’s Disease International (2015). Available at: https://www.alzint.org/u/WorldAlzheimerReport2015.pdf [Accessed July 18, 2023].

[3] Hodson R . Inflammatory bowel disease. Nature. (2016) 540:S97. doi: 10.1038/540S97a 28002398

[4] Ma T Wan M Liu G Zuo X Yang X Yang X . Temporal trends of inflammatory bowel disease burden in China from 1990 to 2030 with comparisons to Japan, South Korea, the European Union, the United States of America, and the world. Clin Epidemiol. (2023) 15:583–99. doi: 10.2147/clep.s402718 PMC10178411 37187768

[5] Cryan JF O'Riordan KJ Cowan CSM Sandhu KV Bastiaanssen TFS Boehme M . The microbiota-gut-brain axis. Physiol Rev. (2019) 99:1877–2013. doi: 10.1152/physrev.00018.2018 31460832

[6] Ghaisas S Maher J Kanthasamy A . Gut microbiome in health and disease: linking the microbiome-gut-brain axis and environmental factors in the pathogenesis of systemic and neurodegenerative diseases. Pharmacol Ther. (2016) 158:52–62. doi: 10.1016/j.pharmthera.2015.11.012 26627987 PMC4747781

[7] Fu P Gao M Yung KKL . Association of intestinal disorders with Parkinson's disease and Alzheimer's disease: A systematic review and meta-analysis. ACS Chem Neurosci. (2020) 11:395–405. doi: 10.1021/acschemneuro.9b00607 31876406

[8] Wang X Wan J Wang M Zhang Y Wu K Yang F . Multiple sclerosis and inflammatory bowel disease: A systematic review and meta-analysis. Ann Clin Trans Neurol. (2022) 9:132–40. doi: 10.1002/acn3.51495 PMC8862424 35092169

[9] Zhu Y Yuan M Liu Y Yang F Chen WZ Xu ZZ . Association between inflammatory bowel diseases and Parkinson's disease: systematic review and meta-analysis. Neural regeneration Res. (2022) 17:344–53. doi: 10.4103/1673-5374.317981 PMC8463981 34269209

[10] Zuin M De Giorgio R Capatti E Boschetti E Zuliani G . Inflammatory bowel disease as a new risk factor for dementia. Aging Clin Exp Res. (2022) 34:1725–8. doi: 10.1007/s40520-022-02076-1 35075587

[11] Page MJ McKenzie JE Bossuyt PM Boutron I Hoffmann TC Mulrow CD . The prisma 2020 statement: an updated guideline for reporting systematic reviews. BMJ (Clinical Res ed). (2021) 372:n71. doi: 10.1136/bmj.n71 PMC8005924 33782057

[12] Stang A . Critical evaluation of the newcastle-ottawa scale for the assessment of the quality of nonrandomized studies in meta-analyses. Eur J Epidemiol. (2010) 25:603–5. doi: 10.1007/s10654-010-9491-z 20652370

[13] Higgins JP Thompson SG . Quantifying heterogeneity in a meta-analysis. Stat Med. (2002) 21:1539–58. doi: 10.1002/sim.1186 12111919

[14] Egger M Smith GD Phillips AN . Meta-analysis: principles and procedures. BMJ (Clinical Res ed). (1997) 315:1533–7. doi: 10.1136/bmj.315.7121.1533 PMC2127925 9432252

[15] Aggarwal M Alkhayyat M Abou Saleh M Sarmini MT Singh A Garg R . Alzheimer disease occurs more frequently in patients with inflammatory bowel disease: insight from a nationwide study. J Clin Gastroenterol. (2022) 57(5):501–507. doi: 10.1097/MCG.0000000000001714 35470286

[16] Kim GH Lee YC Kim TJ Kim ER Hong SN Chang DK . Risk of neurodegenerative diseases in patients with inflammatory bowel disease: A nationwide population-based cohort study. J Crohn's Colitis. (2022) 16:436–43. doi: 10.1093/ecco-jcc/jjab162 34499125

[17] Li X Sundquist J Zöller B Sundquist K . Dementia and Alzheimer's disease risks in patients with autoimmune disorders. Geriatrics gerontology Int. (2018) 18:1350–5. doi: 10.1111/ggi.13488 30044040

[18] Rønnow Sand J Troelsen FS Horváth-Puhó E Henderson VW Sørensen HT Erichsen R . Risk of dementia in patients with inflammatory bowel disease: A danish population-based study. Alimentary Pharmacol Ther. (2022) 56:831–43. doi: 10.1111/apt.17119 PMC9545113 35781292

[19] Vadstrup K Alulis S Borsi A Jørgensen TR Nielsen A Munkholm P . Extraintestinal manifestations and other comorbidities in ulcerative colitis and crohn disease: a danish nationwide registry study 2003-2016. Crohn's colitis 360. (2020) 2(3):otaa070. doi: 10.1093/crocol/otaa070 36776496 PMC9802257

[20] Zhang B Wang HE Bai YM Tsai SJ Su TP Chen TJ . Inflammatory bowel disease is associated with higher dementia risk: A nationwide longitudinal study. Gut. (2021) 70:85–91. doi: 10.1136/gutjnl-2020-320789 32576641

[21] Turner MR Goldacre R Ramagopalan S Talbot K Goldacre MJ . Autoimmune disease preceding amyotrophic lateral sclerosis: an epidemiologic study. Neurology. (2013) 81:1222–5. doi: 10.1212/WNL.0b013e3182a6cc13 PMC3795611 23946298

[22] Bernstein CN Nugent Z Shaffer S Singh H Marrie RA . Comorbidity before and after a Diagnosis of Inflammatory Bowel Disease. Alimentary Pharmacol Ther. (2021) 54:637–51. doi: 10.1111/apt.16444 34156724

[23] Dregan A Chowienczyk P Gulliford MC . Are inflammation and related therapy associated with all-cause dementia in a primary care population? J Alzheimer's Dis. (2015) 46:1039–47. doi: 10.3233/JAD-150171 26402631

[24] Sun Y Geng J Chen X Chen H Wang X Chen J . Association between inflammatory bowel disease and dementia: A longitudinal cohort study. Inflammatory bowel Dis. (2022) 28:1520–6. doi: 10.1093/ibd/izab300 PMC9527613 34849925

[25] Zingel R Bohlken J Kostev K . Association between inflammatory bowel disease and dementia: A retrospective cohort study. J Alzheimer's Dis. (2021) 80:1471–8. doi: 10.3233/JAD-210103 33720902

[26] Avasarala J Guduru Z McLouth CJ Wilburn A Talbert J Sutton P . Use of anti-tnf-A Therapy in Crohn's disease is associated with increased incidence of multiple sclerosis. Multiple sclerosis related Disord. (2021) 51:102942. doi: 10.1016/j.msard.2021.102942 PMC8263493 33933908

[27] Burisch J Jess T Egeberg A . Incidence of immune-mediated inflammatory diseases among patients with inflammatory bowel diseases in Denmark. Clin Gastroenterol AND Hepatol. (2019) 17:2704–+. doi: 10.1016/j.cgh.2019.03.040 30936024

[28] Card TR Langan SM Chu TP . Extra-gastrointestinal manifestations of inflammatory bowel disease may be less common than previously reported. Digestive Dis Sci. (2016) 61:2619–26. doi: 10.1007/s10620-016-4195-1 27193564

[29] Gupta G Gelfand JM Lewis JD . Increased risk for demyelinating diseases in patients with inflammatory bowel disease. Gastroenterology. (2005) 129:819–26. doi: 10.1053/j.gastro.2005.06.022 16143121

[30] Park SW Kim TJ Lee JY Kim ER Hong SN Chang DK . Comorbid immune-mediated diseases in inflammatory bowel disease: A nation-wide population-based study. Alimentary Pharmacol Ther. (2019) 49:165–72. doi: 10.1111/apt.15076 30506945

[31] Villumsen M Aznar S Pakkenberg B Jess T Brudek T . Inflammatory bowel disease increases the risk of Parkinson's disease: A danish nationwide cohort study 1977-2014. Gut. (2019) 68:18–24. doi: 10.1136/gutjnl-2017-315666 29785965

[32] Coates MD Ba DM Liu G Dalessio S Leslie DL Huang X . Revisiting the association between inflammatory bowel disease and Parkinson's disease. Inflammatory bowel Dis. (2022) 28:850–4. doi: 10.1093/ibd/izab175 34259840

[33] Li X Sundquist J Sundquist K . Subsequent risks of parkinson disease in patients with autoimmune and related disorders: A nationwide epidemiological study from Sweden. Neuro-degenerative Dis. (2012) 10:277–84. doi: 10.1159/000333222 22205172

[34] Lin JC Lin CS Hsu CW Lin CL Kao CH . Association between parkinson's disease and inflammatory bowel disease: A nationwide Taiwanese retrospective cohort study. Inflammatory bowel Dis. (2016) 22:1049–55. doi: 10.1097/mib.0000000000000735 26919462

[35] Loosen SH Yaqubi K May P Konrad M Gollop C Luedde T . Association between inflammatory bowel disease and subsequent development of restless legs syndrome and Parkinson's disease: a retrospective cohort study of 35,988 primary care patients in Germany. Life (Basel Switzerland). (2023) 13(4):897. doi: 10.3390/life13040897 37109426 PMC10145108

[36] Park S Kim J Chun J Han K Soh H Kang EA . Patients with inflammatory bowel disease are at an increased risk of Parkinson's disease: a South Korean nationwide population-based study. J Clin Med. (2019) 8(8):1191. doi: 10.3390/jcm8081191 31398905 PMC6723604

[37] Peter I Dubinsky M Bressman S Park A Lu C Chen N . Anti-tumor necrosis factor therapy and incidence of Parkinson disease among patients with inflammatory bowel disease. JAMA Neurol. (2018) 75:939–46. doi: 10.1001/jamaneurol.2018.0605 PMC6142934 29710331

[38] Weimers P Halfvarson J Sachs MC Saunders-Pullman R Ludvigsson JF Peter I . Inflammatory bowel disease and Parkinson's disease: A nationwide Swedish cohort study. Inflammatory bowel Dis. (2019) 25:111–23. doi: 10.1093/ibd/izy190 29788069

[39] Capkun G Dahlke F Lahoz R Nordstrom B Tilson HH Cutter G . Mortality and comorbidities in patients with multiple sclerosis compared with a population without multiple sclerosis: an observational study using the us department of defense administrative claims database. Multiple sclerosis related Disord. (2015) 4:546–54. doi: 10.1016/j.msard.2015.08.005 26590661

[40] Nielsen NM Frisch M Rostgaard K Wohlfahrt J Hjalgrim H Koch-Henriksen N . Autoimmune diseases in patients with multiple sclerosis and their first-degree relatives: A nationwide cohort study in Denmark. Multiple sclerosis (Houndmills Basingstoke England). (2008) 14:823–9. doi: 10.1177/1352458508088936 18573841

[41] Roshanisefat H Bahmanyar S Hillert J Olsson T Montgomery S . Shared genetic factors may not explain the raised risk of comorbid inflammatory diseases in multiple sclerosis. Multiple sclerosis (Houndmills Basingstoke England). (2012) 18:1430–6. doi: 10.1177/1352458512438240 22419672

[42] Duval S Tweedie R . Trim and fill: A simple funnel-plot-based method of testing and adjusting for publication bias in meta-analysis. Biometrics. (2000) 56:455–63. doi: 10.1111/j.0006-341X.2000.00455.x 10877304

[43] Hou Y Dan X Babbar M Wei Y Hasselbalch SG Croteau DL . Ageing as a risk factor for neurodegenerative disease. Nat Rev Neurol. (2019) 15:565–81. doi: 10.1038/s41582-019-0244-7 31501588

[44] Bhat R Crowe EP Bitto A Moh M Katsetos CD Garcia FU . Astrocyte senescence as a component of Alzheimer's disease. PloS One. (2012) 7:e45069. doi: 10.1371/journal.pone.0045069 22984612 PMC3440417

[45] Boccardi V Pelini L Ercolani S Ruggiero C Mecocci P . From cellular senescence to Alzheimer's disease: the role of telomere shortening. Ageing Res Rev. (2015) 22:1–8. doi: 10.1016/j.arr.2015.04.003 25896211

[46] Chinta SJ Woods G Demaria M Rane A Zou Y McQuade A . Cellular senescence is induced by the environmental neurotoxin paraquat and contributes to neuropathology linked to parkinson's disease. Cell Rep. (2018) 22:930–40. doi: 10.1016/j.celrep.2017.12.092 PMC5806534 29386135

[47] Turnquist C Horikawa I Foran E Major EO Vojtesek B Lane DP . P53 isoforms regulate astrocyte-mediated neuroprotection and neurodegeneration. Cell Death differentiation. (2016) 23:1515–28. doi: 10.1038/cdd.2016.37 PMC5072428 27104929

[48] Madore C Yin Z Leibowitz J Butovsky O . Microglia, lifestyle stress, and neurodegeneration. Immunity. (2020) 52:222–40. doi: 10.1016/j.immuni.2019.12.003 PMC7234821 31924476

[49] Ananthakrishnan AN Bernstein CN Iliopoulos D Macpherson A Neurath MF Ali RAR . Environmental triggers in ibd: A review of progress and evidence. Nat Rev Gastroenterol Hepatol. (2018) 15:39–49. doi: 10.1038/nrgastro.2017.136 29018271

[50] Chen Y Cui W Li X Yang H . Interaction between commensal bacteria, immune response and the intestinal barrier in inflammatory bowel disease. Front Immunol. (2021) 12:761981. doi: 10.3389/fimmu.2021.761981 34858414 PMC8632219

[51] Li Y Chen Y Jiang L Zhang J Tong X Chen D . Intestinal inflammation and Parkinson's disease. Aging Dis. (2021) 12:2052–68. doi: 10.14336/ad.2021.0418 PMC8612622 34881085

[52] Jia W Rajani C Kaddurah-Daouk R Li H . Expert insights: the potential role of the gut microbiome-bile acid-brain axis in the development and progression of Alzheimer's disease and hepatic encephalopathy. Medicinal Res Rev. (2020) 40:1496–507. doi: 10.1002/med.21653 31808182

[53] Wang D Zhang X Du H . Inflammatory bowel disease: A potential pathogenic factor of Alzheimer's disease. Prog Neuropsychopharmacol Biol Psychiatry. (2022) 119:110610. doi: 10.1016/j.pnpbp.2022.110610 35908596

[54] Mayer EA Nance K Chen S . The gut-brain axis. Annu Rev Med. (2022) 73:439–53. doi: 10.1146/annurev-med-042320-014032 34669431

[55] Mulak A . Bile acids as key modulators of the brain-gut-microbiota axis in Alzheimer's disease. J Alzheimer's Dis JAD. (2021) 84:461–77. doi: 10.3233/jad-210608 PMC8673511 34569953

[56] Ni J Wu GD Albenberg L Tomov VT . Gut microbiota and ibd: causation or correlation? Nat Rev Gastroenterol Hepatol. (2017) 14:573–84. doi: 10.1038/nrgastro.2017.88 PMC5880536 28743984

[57] Zhuang ZQ Shen LL Li WW Fu X Zeng F Gui L . Gut microbiota is altered in patients with Alzheimer's disease. J Alzheimer's Dis JAD. (2018) 63:1337–46. doi: 10.3233/jad-180176 29758946

[58] Zeng Q Shen J Chen K Zhou J Liao Q Lu K . The alteration of gut microbiome and metabolism in amyotrophic lateral sclerosis patients. Sci Rep. (2020) 10:12998. doi: 10.1038/s41598-020-69845-8 32747678 PMC7398913

[59] Hill-Burns EM Debelius JW Morton JT Wissemann WT Lewis MR Wallen ZD . Parkinson's disease and Parkinson's disease medications have distinct signatures of the gut microbiome. Movement Disord Off J Movement Disord Soc. (2017) 32:739–49. doi: 10.1002/mds.26942 PMC5469442 28195358

[60] Bedarf JR Hildebrand F Coelho LP Sunagawa S Bahram M Goeser F . Functional implications of microbial and viral gut metagenome changes in early stage L-dopa-naïve Parkinson's disease patients. Genome Med. (2017) 9:39. doi: 10.1186/s13073-017-0428-y 28449715 PMC5408370

[61] Kang X Ploner A Wang Y Ludvigsson JF Williams DM Pedersen NL . Genetic overlap between Parkinson’s disease and inflammatory bowel disease. Brain Commun. (2023) 5(1):fcad002. doi: 10.1093/braincomms/fcad002 36687396 PMC9847552

[62] Brudek T . Inflammatory bowel diseases and Parkinson's disease. J Parkinson's Dis. (2019) 9:S331–s44. doi: 10.3233/jpd-191729 PMC6839501 31609699

[63] Hui KY Fernandez-Hernandez H Hu J Schaffner A Pankratz N Hsu NY . Functional variants in the lrrk2 gene confer shared effects on risk for Crohn's disease and Parkinson's disease. Sci Trans Med. (2018) 10(423):eaai7795. doi: 10.1126/scitranslmed.aai7795 PMC6028002 29321258

[64] Di Bona D Rizzo C Bonaventura G Candore G Caruso C . Association between interleukin-10 polymorphisms and Alzheimer's disease: A systematic review and meta-analysis. J Alzheimer's Dis JAD. (2012) 29:751–9. doi: 10.3233/jad-2012-111838 22356904

[65] Braak H de Vos RA Bohl J Del Tredici K . Gastric alpha-synuclein immunoreactive inclusions in Meissner's and Auerbach's plexuses in cases staged for Parkinson's disease-related brain pathology. Neurosci Lett. (2006) 396:67–72. doi: 10.1016/j.neulet.2005.11.012 16330147

[66] Miehsler W Reinisch W Valic E Osterode W Tillinger W Feichtenschlager T . Is inflammatory bowel disease an independent and disease specific risk factor for thromboembolism? Gut. (2004) 53:542–8. doi: 10.1136/gut.2003.025411 PMC1773996 15016749

[67] Kappelman MD Horvath-Puho E Sandler RS Rubin DT Ullman TA Pedersen L . Thromboembolic risk among danish children and adults with inflammatory bowel diseases: A population-based nationwide study. Gut. (2011) 60:937–43. doi: 10.1136/gut.2010.228585 21339206

[68] Nguyen GC Sam J . Rising prevalence of venous thromboembolism and its impact on mortality among hospitalized inflammatory bowel disease patients. Am J Gastroenterol. (2008) 103:2272–80. doi: 10.1111/ajg.2008.103.issue-9 18684186

[69] Rao M Gershon MD . The bowel and beyond: the enteric nervous system in neurological disorders. Nat Rev Gastroenterol Hepatol. (2016) 13:517–28. doi: 10.1038/nrgastro.2016.107 PMC5005185 27435372

[70] Neunlist M Van Landeghem L Bourreille A Savidge T . Neuro-glial crosstalk in inflammatory bowel disease. J Intern Med. (2008) 263:577–83. doi: 10.1111/j.1365-2796.2008.01963.x 18479256

[71] Ferreira-Vieira TH Guimaraes IM Silva FR Ribeiro FM . Alzheimer's disease: targeting the cholinergic system. Curr neuropharmacology. (2016) 14:101–15. doi: 10.2174/1570159x13666150716165726 PMC4787279 26813123

[72] Marino BLB de Souza LR Sousa KPA Ferreira JV Padilha EC da Silva C . Parkinson's disease: A review from pathophysiology to treatment. Mini Rev medicinal Chem. (2020) 20:754–67. doi: 10.2174/1389557519666191104110908 31686637

[73] Luchetti S Huitinga I Swaab DF . Neurosteroid and gaba-a receptor alterations in Alzheimer's disease, Parkinson's disease and multiple sclerosis. Neuroscience. (2011) 191:6–21. doi: 10.1016/j.neuroscience.2011.04.010 21514366

[74] Czapski GA Strosznajder JB . Glutamate and gaba in microglia-neuron cross-talk in Alzheimer's disease. Int J Mol Sci. (2021) 22(21):11677. doi: 10.3390/ijms222111677 34769106 PMC8584169

[75] Filpa V Moro E Protasoni M Crema F Frigo G Giaroni C . Role of glutamatergic neurotransmission in the enteric nervous system and brain-gut axis in health and disease. Neuropharmacology. (2016) 111:14–33. doi: 10.1016/j.neuropharm.2016.08.024 27561972

[76] Kaltsonoudis E Voulgari PV Konitsiotis S Drosos AA . Demyelination and other neurological adverse events after anti-tnf therapy. Autoimmun Rev. (2014) 13:54–8. doi: 10.1016/j.autrev.2013.09.002 24035809

