# Supplementary material for: The two-directional prospective association between inflammatory bowel disease and neurodegenerative disorders: a systematic review and meta-analysis based on longitudinal studies
Source: Front Immunol. 2024 Apr 24;15:1325908. doi: 10.3389/fimmu.2024.1325908 (PMC11076839; doi:10.3389/fimmu.2024.1325908)
Supplement: Supplementary file 1 [file DataSheet_1.doc]

**Supplementary Figures and Tables**

# Supplementary tables

| **study** | **disease** | **Country** | **Age(years)** | **Study period** | **follow-up time** | **effect estimate, 95%CI** |  | **NDD** | **Concomitant disease** | **Non-NDD** | **Concomitant disease** | **NOS** |
| --- | --- | --- | --- | --- | --- | --- | --- | --- | --- | --- | --- | --- |
| Capkun,2015^(39)^ | MS | USA | 46.0±11.7^†^ | 2006-2011 | 1518±638 days | UC:1.98 (1.69–2.33)* | HR | MS:15,684 | UC:210 | 78,420 | UC(496) | 8 |
| Nielsen,2008^(40)^ | MS | Danmark | NA | 1977-2004 | NA | CD:0.7 (0.3–1.5)* UC:2.0 (1.4–2.8)* | SIR | MS:12,403 | CD:6 UC:29 | NA | expCD(9.2) expUC(14.9) | 8 |
| Roshanisefat,2012^(41)^ | MS | Sweden | 35.1^‡^ | 1964-2005 | NA | CD:1.70 (1.23–2.34) UC:1.81 (1.38–2.39) | HR | MS:20,276 | CD:93 UC:113 | 203,951 | CD(669) UC(819) | 8 |

MS: multiple sclerosis ; NDD: neurodegenerative disease ; IBD: inflammatory bowel disease; CD: Crohn's disease; UC: ulcerative colitis; HR: hazard ratio; SIR: standardized risk ratio; IRR: incidence rate ratio

^†^ mean ± SD ^‡^ mean * unadjusted effect estimate

**Supplementary Table 1 : Characteristics of 3 studies exploring the risk of IBD in patients with neurodegenerative disease**

**Supplementary Table 2 : Newcastle-Ottawa Quality Assessment Scale**

|  | effect estimate （95%CI） | Test for overall effect | No of studies | Heterogeneity I² | Egger’s test |
| --- | --- | --- | --- | --- | --- |
| AD≥50^(16,17,20)^ | 1.44 (1.04-2.00) | Z=2.17, p=0.030 | 4 | 89.7% | 0.308 |
| AD<50^(18)^ | 1.02 (0.85-1.22) | Z=0.20, p=0.845 | 2 | 71.6% | — |
|  |  |  |  |  |  |
| Dementia≥50^(17,20,24,25)^ | 1.34 (1.11-1.62) | Z=3.08, p=0.002 | 5 | 84.5% | 0.113 |
| Dementia<50^(18,22,23)^ | 1.14 (1.05-1.22) | Z=3.30, p=0.001 | 5 | 37.2% | 0.011 |
|  |  |  |  |  |  |
| MS≥50 | — | — | — | — | — |
| MS<50^(26-30)^ | 2.07 (1.42-3.02) | Z=3.78, p<0.0001 | 6 | 76.5% | 0.963 |
|  |  |  |  |  |  |
| PD≥50^(16,35,37)^ | 1.25 (1.05-1.49) | Z=2.47, p=0.014 | 4 | 62.5% | 0.823 |
| PD<50^(32,34,36,38)^ | 1.23 (0.89-1.70) | Z=1.28, p=0.202 | 4 | 84.0% | 0.945 |

**Supplementary Table 3 : Subgroup analyses—association between IBD and neurodegenerative diseases, stratified by age**

|  | effect estimate （95%CI） | Test for overall effect | No of studies | Heterogeneity I² | Egger’s test |
| --- | --- | --- | --- | --- | --- |
| AD-CD^(15-20)^ | 1.69 (0.87-3.28) | Z=1.56, p=0.119 | 6 | 98.8% | 0.089 |
| AD-UC^(15-20)^ | 1.52 (0.99-2.32) | Z=2.31, p=0.021 | 6 | 99.7% | 0.257 |
|  |  |  |  |  |  |
| Dementia-CD^(17,18,20,22-25)^ | 1.27 (1.13-1.43) | Z=4.03, p<0.0001 | 7 | 51.8% | 0.042 |
| Dementia-UC^(17,18,20,22-25)^ | 1.25 (1.14-1.36) | Z=3.08, p=0.002 | 7 | 74.1% | 0.145 |
|  |  |  |  |  |  |
| MS-CD^(27,29,30)^ | 2.19 (1.21-1.83) | Z=2.66, p=0.008 | 3 | 4.9%* | 0.256 |
| MS-UC^(27,29,30)^ | 1.96 (1.25-3.06) | Z=2.94, p=0.003 | 3 | 0%* | 0.888 |
|  |  |  |  |  |  |
| PD-CD^(16,19,32-38)^ | 1.21 (1.03-1.41) | Z=2.38, p=0.017 | 9 | 32.5% | 0.475 |
| PD-UC^(16,19,32-38)^ | 1.23 (1.02-1.48) | Z=2.11, p=0.034 | 9 | 75.2% | 0.477 |

*** Fixed-effects model was used because of the low heterogeneity.**

**Supplementary Table 4 : Subgroup analyses—association between IBD and neurodegenerative diseases, stratified by IBD subgroup**

|  | effect estimate （95%CI） | Test for overall effect | No of studies | Heterogeneity I² | | Egger’s test |
| --- | --- | --- | --- | --- | --- | --- |
| AD-Europe^(17-19)^ | 1.07 (1.00-1.14) | Z=2.05, p=0.040 | 6 | 0.0% | 0.688 | |
| AD-America^(15)^ | 2.30 (2.10-2.51) | Z=18.31, p<0.001 | 1 | — | — | |
| AD-Asia^(16,20)^ | 2.58 (0.49-13.52) | Z=1.12, p=0.262 | 2 | 96.4% | — | |
|  |  |  |  |  |  | |
| Dementia-Europe^(17,18,23-25)^ | 1.16 (1.10-1.24) | Z=5.01, p<0.001 | 8 | 44.2% | 0.030 | |
| Dementia-America^(22)^ | 1.18 (1.02-1.36) | Z=2.26, p=0.024 | 1 | — | — | |
| Dementia-Asia^(20)^ | 2.54 (1.91-3.37) | Z=6.44, p<0.001 | 1 | — | — | |
|  |  |  |  |  |  | |
| MS-Eupore^(27-29,31)^ | 2.58 (2.14-3.10) | Z=10.01, p<0.001 | 4 | 13.4%* | 0.345 | |
| MS-America^(26)^ | 1.32 (1.02-1.70) | Z=2.15, p=0.032 | 1 | — | — | |
| MS-Asia^(30)^ | 2.89 (1.01-8.30) | Z=1.97, p=0.049 | 1 | — | — | |
|  |  |  |  |  |  | |
| PD-Europe^(19,31,33,35,38)^ | 1.12 (0.97-1.30) | Z=1.54, p=0.125 | 8 | 58.6% | 0.490 | |
| PD-America^(32,37)^ | 1.20 (0.98-1.48) | Z=1.76, p=0.078 | 2 | 40.2% | — | |
| PD-Asia^(16,34,36)^ | 1.56 (1.30-1.87) | Z=4.85, p<0.001 | 3 | 41.2% | 0.164 | |

***Fixed-effects model was used because of the low heterogeneity.**

**Supplementary Table 5: Subgroup analyses—association between IBD and neurodegenerative diseases, stratified by study country**

|  | effect estimate （95%CI） | Test for overall effect | No of studies | Heterogeneity   I² | Egger’s test |
| --- | --- | --- | --- | --- | --- |
| AD-adjusted^(15,16,18,20)^ | 1.59 (1.08-2.34) | Z=2.35, p=0.019 | 5 | 98.2% | 0.794 |
| AD-unadjusted^(17,19)^ | 1.09 (0.95-1.24) | Z=1.25, p=0.211 | 4 | 0.0% | 0.907 |
|  |  |  |  |  |  |
| Dementia-adjusted^(18,20,23,24)^ | 1.32 (1.11-1.56) | Z=3.14, p=0.002 | 6 | 87.0% | 0.095 |
| Dementia-unadjusted^(17,22,25)^ | 1.21 (1.14-1.29) | Z=6.11, p<0.0001 | 4 | 0.0% | 0.099 |
|  |  |  |  |  |  |
| MS-adjusted^(26-28)^ | 1.87 (1.09-3.23) | Z=2.26, p=0.024 | 3 | 90.2% | 0.797 |
| MS-unadjusted^(29,30)^ | 2.48 (1.56-3.95) | Z=3.83, p<0.0001 | 3 | 0.0% | 0.774 |
|  |  |  |  |  |  |
| PD-adjusted^(16,31-34,36-38)^ | 1.24 (1.08-1.42) | Z=3.11, p=0.002 | 9 | 71.6% | 0.662 |
| PD-unadjusted^(19,35)^ | 1.22 (0.96-1.55) | Z=1.63, p=0.103 | 4 | 54.2% | 0.684 |

**Supplementary Table 6 : Subgroup analyses—association between IBD and neurodegenerative diseases, stratified by covariate adjustment**

# Supplementary Figures





**Supplementary Figure 1 The PRISMA flow diagram for the search and selection processes of the meta-analysis.**


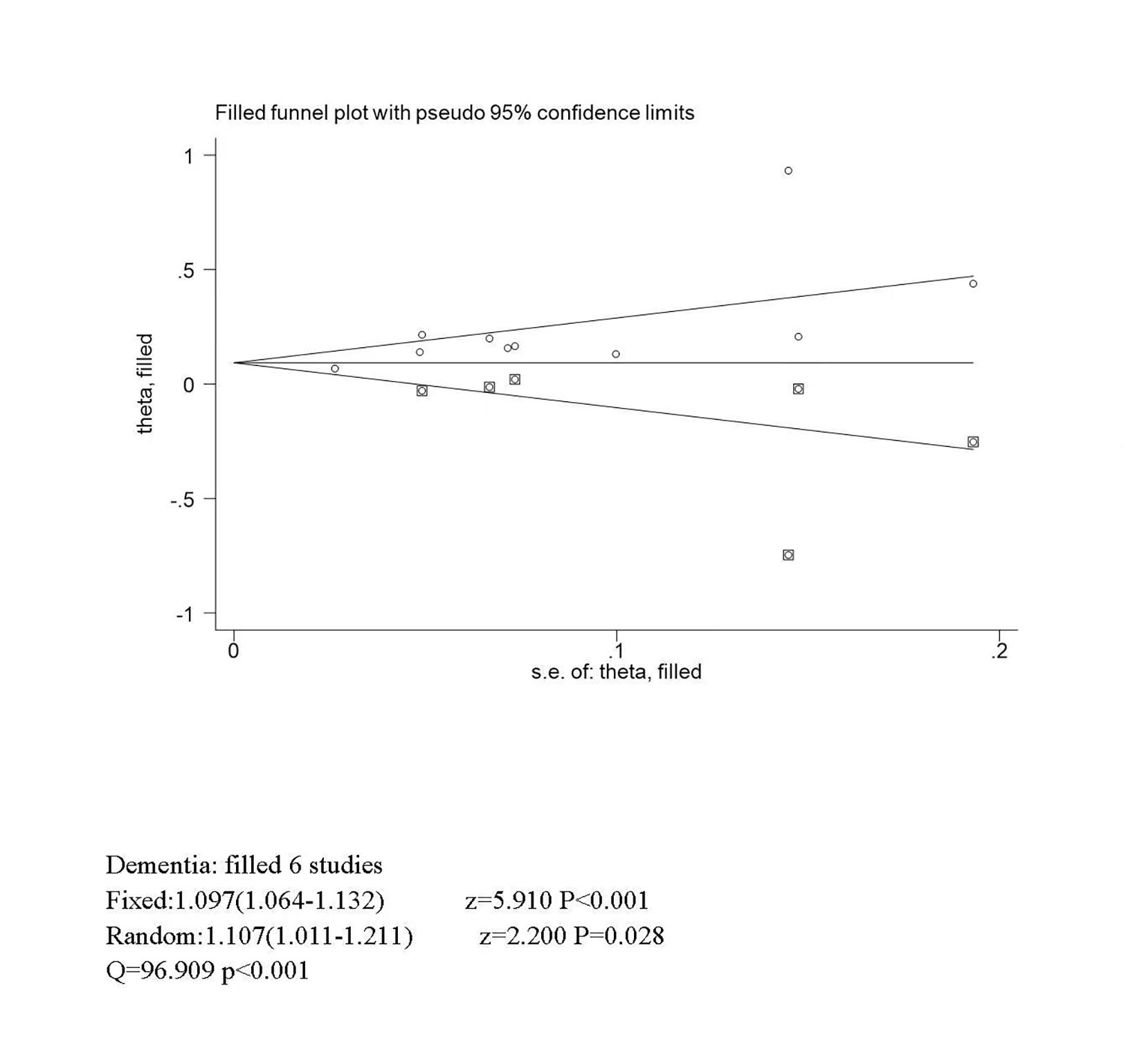


**Supplementary Figure 2:Trim-and-fill method for IBD and dementia**
