# Supplementary material for: The two-directional prospective association between inflammatory bowel disease and neurodegenerative disorders: a systematic review and meta-analysis based on longitudinal studies
Source: Front Immunol. 2024 Apr 24;15:1325908. doi: 10.3389/fimmu.2024.1325908 (PMC11076839; doi:10.3389/fimmu.2024.1325908)
Supplement: Supplementary file 2 [file DataSheet_2.doc]

**Search strategy**

****Up to June 18 2023****

****PubMed****

#1:

"Inflammatory bowel diseases"[MeSH Terms] OR "inflammatory bowel disease*"[Title/Abstract] OR "inflammatory bowel disorder*"[Title/Abstract] OR "ulcerative colitis"[Title/Abstract] OR "proctocolitis"[MeSH Terms] OR "idiopathic proctocolitis"[Title/Abstract] OR "ulcerative proctocolitis"[Title/Abstract] OR "colitis Gravis"[Title/Abstract] OR "crohn*"[Title/Abstract] OR "regional enteritis"[Title/Abstract] OR "ileitis"[Title/Abstract] OR "IBD"[Title/Abstract]

139,982

#2:

"Neurocognitive disorders"[MeSH Terms] OR "neurological disorder*"[Title/Abstract] OR "neurodegenerative*"[Title/Abstract] OR "alzheimer*"[Title/Abstract] OR "parkinson*"[Title/Abstract] OR "huntington*"[Title/Abstract] OR "Amyotrophic lateral sclerosis"[Title/Abstract] OR "Multiple sclerosis"[Title/Abstract] OR "lewy body disease*"[Title/Abstract] OR "Frontotemporal Lobar degeneration"[Title/Abstract] OR "progressive supranuclear palsy"[Title/Abstract] OR "spinal muscular atrophy"[Title/Abstract] OR "dementia"[Title/Abstract] OR "cognitive impairment"[Title/Abstract]

732,660

#3:

"cohort studies"[MeSH Terms] OR "cohort"[Title/Abstract] OR "longitudinal studies"[MeSH Terms] OR "longitudinal"[Title/Abstract] OR "follow up studies"[MeSH Terms] OR "follow-up"[Title/Abstract] OR "prospective"[Title/Abstract] OR "registration system"[Title/Abstract] OR "incidence"[Title/Abstract] OR "Cox"[Title/Abstract] OR "relative risk"[Title/Abstract] OR "hazard risk"[Title/Abstract]

4,364,140

**#1 AND #2 AND #3 406**

**Web of science**

#1:

TS=(“Neurocognitive disorder*”) OR TS=(“neurological disorder*”) OR TS=("neurodegenerative") OR TS=(“alzheimer*”) OR TS=(“parkinson*”) OR TS=("huntington*")OR TS=("Amyotrophic lateral sclerosis") OR TS=("Multiple sclerosis") OR TS=("lewy body disease*") OR TS=("Frontotemporal Lobar degeneration") OR TS=("progressive supranuclear palsy") OR TS=("spinal muscular atrophy") OR TS=("dementia") OR TS=("cognitive impairment")

1,165,204

#2:

TS=(“inflammatory bowel disease*”) OR TS=(“inflammatory bowel disorder*") OR TS=("ulcerative colitis") OR TS=("proctocolitis") OR TS=("colitis Gravis") OR TS=("crohn*") OR TS=("regional enteritis") OR TS=("ileitis") OR TS=("IBD")

[254,368](https://www.webofscience.com/wos/alldb/summary/9a81f933-d240-4987-b346-8f0f2094842f-9247d9d3/relevance/1)

#3:

TS=("cohort") OR TS=("longitudinal") OR TS=("follow-up") OR TS=("prospective") OR TS=("registration system") OR TS=("incidence") OR TS=("Cox") OR TS=("relative risk") OR TS=("hazard risk")

[5,605,512](https://www.webofscience.com/wos/alldb/summary/7cbd1ec6-d584-4b9e-b56b-a552bd605c81-9247dda4/relevance/1)

**#1 AND #2 AND #3** [**795**](https://www.webofscience.com/wos/alldb/summary/9a37b2ec-a353-4ad5-8a2c-0d1154ce18c9-9247df61/relevance/1)

**Embase**

#1:

'inflammatory bowel disease'/exp OR 'inflammatory bowel disease' OR 'inflammatory bowel disease*':ab,ti OR 'inflammatory bowel disorder*':ab,ti OR 'ulcerative colitis'/exp OR 'ulcerative colitis' OR colitis:ab,ti OR proctocolitis:ab,ti OR 'colitis gravis':ab,ti OR 'crohn disease'/exp OR 'crohn disease' OR 'regional enteritis':ab,ti OR 'regional enterocolitis':ab,ti OR 'crohn*':ab,ti OR 'ileitis':ab,ti OR ibd:ab,ti

277,167

#2:

'degenerative disease'/exp OR 'neurodegenerative':ab,ti OR 'neurocognitive disorder*':ab,ti OR 'neurological disorder*':ab,ti OR 'alzheimer*':ab,ti OR 'parkinson*':ab,ti OR 'huntington*':ab,ti OR 'amyotrophic lateral sclerosis':ab,ti OR 'multiple sclerosis':ab,ti OR 'lewy body disease*':ab,ti OR 'frontotemporal lobar degeneration*':ab,ti OR 'progressive supranuclear palsy':ab,ti OR 'spinal muscular atrophy':ab,ti OR 'dementia':ab,ti OR 'cognitive impairment':ab,ti

[1,230,484](https://www.embase.com/)

#3

'cohort analysis'/exp OR ‘cohort’:ab,ti OR ‘Longitudinal study’/exp OR ‘longitudinal’:ab,ti OR ‘follow up’/exp OR ‘follow-up’:ab,ti OR ‘prospective’:ab,ti OR ‘registration system’:ab,ti OR ‘incidence’:ab,ti OR ‘Cox’:ab,ti OR ‘relative risk’:ab,ti OR ‘hazard risk’:ab,ti

[5,633,151](https://www.embase.com/)

**#1 AND #2 AND #3 1392**

**Psyclnfo**
#1：

AB ("inflammatory bowel disease*") OR ("inflammatory bowel disorder*") OR ("ulcerative colitis") OR ("proctocolitis") OR ("colitis") OR ("crohn*") OR ("regional enteritis") OR ("ileitis") OR ("IBD")

2,193

#2：

AB ("Neurocognitive disorder*") OR ("neurological disorder*") OR ("neurodegenerative") OR ("alzheimer*") OR ("parkinson*") OR ("huntington*") OR ("Amyotrophic lateral sclerosis") OR ("Multiple sclerosis") OR ("lewy body disease*") OR ("Frontotemporal Lobar degeneration") OR ("progressive supranuclear palsy") OR ("spinal muscular atrophy") OR ("dementia") OR ("cognitive impairment")

193,909

**#1and#2 174**

**Cochrane**

ID Search Hits

#1 MeSH descriptor: [Inflammatory Bowel Diseases] explode all trees 4,811

#2 (""inflammatory bowel NEXT disease*""):ti,ab,kw 4,106

#3 (""inflammatory bowel NEXT disorder*""):ti,ab,kw 37

#4 MeSH descriptor: [Proctocolitis] explode all trees 32

#5 ("ulcerative colitis"):ti,ab,kw 5,921

#6 ("idiopathic proctocolitis"):ti,ab,kw 2

#7 ("ulcerative proctocolitis"):ti,ab,kw 2

#8 ("colitis Gravis"):ti,ab,kw 1

#9 ("crohn*"):ti,ab,kw 5,684

#10 ("regional enteritis"):ti,ab,kw 44

#11 ("ileitis"):ti,ab,kw 179

#12 ("IBD"):ti,ab,kw 2,345

#13 #1or#2or#3or#4or#5or#6or#7or#8or#9or#10or#11or#12 12,410

#14 (""neurological NEXT disorder*""):ti,ab,kw 2,002

#15 ("neurodegenerative*"):ti,ab,kw 2,077

#16 MeSH descriptor: [Neurodegenerative Diseases] explode all trees 14,614

#17 ("alzheimer*"):ti,ab,kw 13,355

#18 ("parkinson*"):ti,ab,kw 12,302

#19 ("huntington*"):ti,ab,kw 789

#20 ("Amyotrophic lateral sclerosis"):ti,ab,kw 1,575

#21 ("Multiple sclerosis"):ti,ab,kw 11,989

#22 (""lewy body NEXT disease*""):ti,ab,kw 280

#23 ("Frontotemporal Lobar degeneration"):ti,ab,kw 40

#24 ("progressive supranuclear palsy"):ti,ab,kw 229

#25 ("spinal muscular atrophy"):ti,ab,kw 288

#26 ("dementia"):ti,ab,kw 16,231

#27 ("cognitive impairment"):ti,ab,kw 11,298

#28 #14or#15or#16or#17or#18or#19or#20or#21or#22or#23or#24or#25or#26or#27 58,857

#29 #13and#28 169

**166 Trials 2Cochrane Reviews 1Editorals**
